# Synthesis of the calcilytic ligand NPS 2143

**DOI:** 10.3762/bjoc.9.154

**Published:** 2013-07-09

**Authors:** Henrik Johansson, Thomas Cailly, Alex Rojas Bie Thomsen, Hans Bräuner-Osborne, Daniel Sejer Pedersen

**Affiliations:** 1 Department of Drug Design and Pharmacology, University of Copenhagen, Universitetsparken 2, DK-2100 Copenhagen, Denmark; 2 Université de Caen Basse-Normandie, CERMN (EA 4258 - FR CNRS 3038 INC3M - SF 4206 ICORE) UFR des Sciences Pharmaceutiques, Bd Becquerel, F-14032 Caen, France

**Keywords:** epoxides, GPCR, NPS 2143, nucleophilic aromatic substitution, pyrylium chemistry

## Abstract

(*R*)-**3** (NPS 2143) is a negative allosteric modulator of the human calcium-sensing receptor (CaSR) and as such represents an important pharmacological tool compound for studying the CaSR. Herein, we disclose for the first time a complete experimental description, detailed characterisation and assessment of enantiomeric purity for (*R*)-**3**. An efficient, reproducible and scalable synthesis of (*R*)-**3** that requires a minimum of chromatographic purification steps is presented. (*R*)-**3** was obtained in excellent optical purity (er > 99:1) as demonstrated by chiral HPLC and the pharmacological profile for (*R*)-**3** is in full accordance with that reported in the literature.

## Introduction

**Figure 1:**
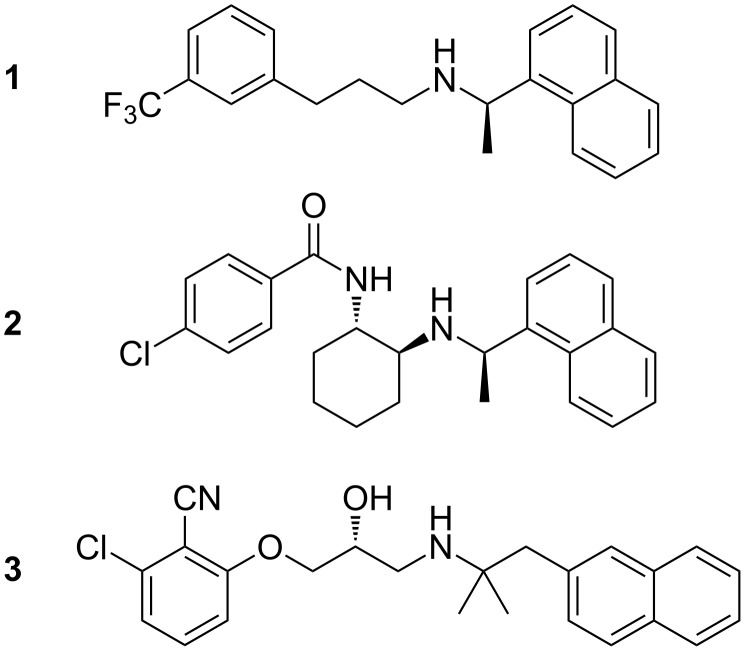
Marketed calcium-sensing receptor agonist cinacalcet (**1**), and CaSR antagonists Calhex 231 (**2**) and NPS 2143 ((*R*)-**3**).

The first G-protein-coupled receptors (GPCRs) were identified more than 30 years ago and have since grown to become the largest class of membrane-bound receptors and the single most common target type in drug discovery and therapy [1–2]. Excluding olfactory receptors there are 390 genes encoding GPCRs in the human genome, divided into three major classes (A, B and C) of which the rhodopsin-like class A is the largest and most diverse, and which has been well-studied and subject to drug targeting [3]. GPCR class C is much smaller and comprises only 22 known receptors including eight metabotropic glutamate (mGlu) receptors, two γ-aminobutyric acid type B (GABA_B_) receptors, three taste (TR) receptors, the GPRC6A receptor, the calcium-sensing receptor (CaSR), and seven orphan receptors [4]. Class C GPCRs have proven viable as drug targets, exemplified by the marketed GABA_B_ agonist baclofen, and by cinacalcet (**1**), a positive allosteric modulator on the CaSR (**1**, Figure 1) [5].

The CaSR is the main regulator of serum calcium homeostasis and plays a central role in several cellular processes such as secretion and regulation of peptide hormones (most importantly parathyroid hormone), ion-channel activity and apoptosis in certain cell types [6–8]. The CaSR is involved in several calcium-regulation-related diseases including various types of hypo- and hyperparathyroidism, and its pronounced regulation of parathyroid hormone secretion makes it an interesting target for drug discovery [9]. Several potent CaSR modulators have been reported over the past decade, including the negative allosteric modulators (calcilytics) **2** [10] and (*R*)-**3** (Figure 1) [11–12].

To facilitate our pharmacological research program on the CaSR we required calcilytic (*R*)-**3** in quantities of several grams as a pharmacological tool compound. We quickly discovered that the published [12–14] experimental details and characterisation for ligand (*R*)-**3** were unsatisfactory, and we were unable to reproduce several of the reported synthetic procedures. Moreover, we were surprised to discover the lack of assessment of optical purity for (*R*)-**3.** Considering that the *R*-enantiomer of (*R*)-**3** and related aminoalcohols display both significantly higher potency and target selectivity than their *S*-enantiomers, we believe that this is a critical shortcoming in the previously reported syntheses [13–14].

Herein, we wish to report a detailed, reproducible and scalable synthesis for racemic and enantiopure calcilytic agent (*R*)-**3**, including detailed spectroscopic, chromatographic and pharmacological characterisation.

## Results and Discussion

**Scheme 1:**
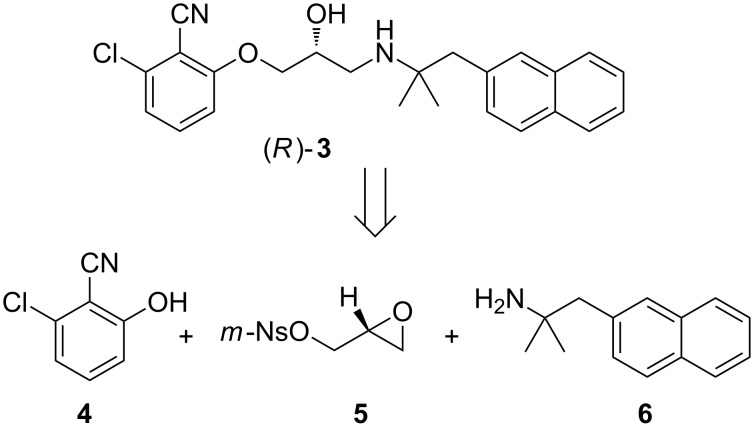
Strategy for assembling (*R*)-**3** from fragments **4**, **5** and **6**. *m*-Ns = *m*-nitrobenzenesulfonyl.

We decided to synthesise (*R*)-**3** using the same overall strategy published by others employing the three fragments depicted in Scheme 1. Similar to a previously reported synthesis of (*R*)-**3** [13], we decided to activate epoxide **5** as the *m*-nosyl derivative that has been shown to minimise racemisation during epoxide ring opening [15].

**Scheme 2:**
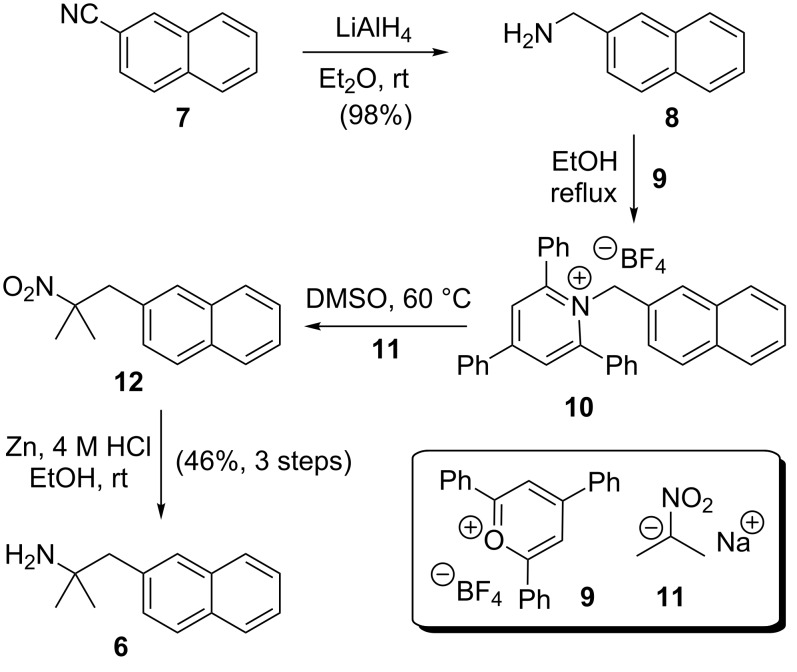
Synthesis of amine building block **6** by using Katritzky’s pyrylium chemistry [16].

The synthesis of amine **6** was accomplished from commercially available 2-cyanonaphthalene (**7**) in four steps (Scheme 2). Reduction of **7** was easily achieved on multigram scale by using LiAlH_4_ to provide amine **8** in excellent yield and purity after work-up. According to the method of Katritzky et al. [16] amine **8** was activated as the corresponding pyridinium salt **10** upon treatment with triphenylpyrylium salt **9** in ethanol under reflux. Subsequently, the crude pyridinium salt **10** was exposed to the sodium salt of 2-nitropropane (**11**) to give nitro compound **12**. Reduction of the nitro group in **12** by catalytic hydrogenation at atmospheric pressure to produce amine **6** has previously been described by Kamal and Chouhan [17] but was unsuccessful in our hands, returning the starting material under a variety of conditions. Eventually, the reduction was realised using zinc and HCl in ethanol to give amine **6** in good yield after chromatographic purification.

**Scheme 3:**
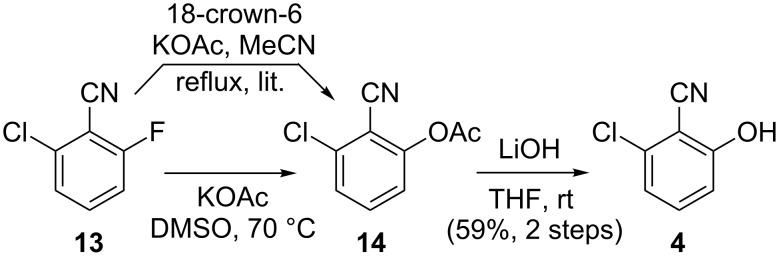
Synthesis of phenol **4** from commercially available aryl fluoride **13**.

Next we turned our attention to the synthesis of phenol **4** (Scheme 3). Previously, **4** has been synthesised by nucleophilic aromatic substitution on commercially available aryl fluoride **13** using crown ether and potassium acetate in acetonitrile [14]. Unfortunately, we found it difficult to drive the reaction to completion using the published method, obtaining only low yields of acetate **14** even under forcing conditions. However, performing the reaction in DMSO with potassium acetate, conditions that we have previously applied successfully [18], gave a clean reaction and high yield of acetate **14**. Basic hydrolysis of crude acetate **14** produced phenol **4** of high purity and in good yield after aqueous work-up.

**Scheme 4:**
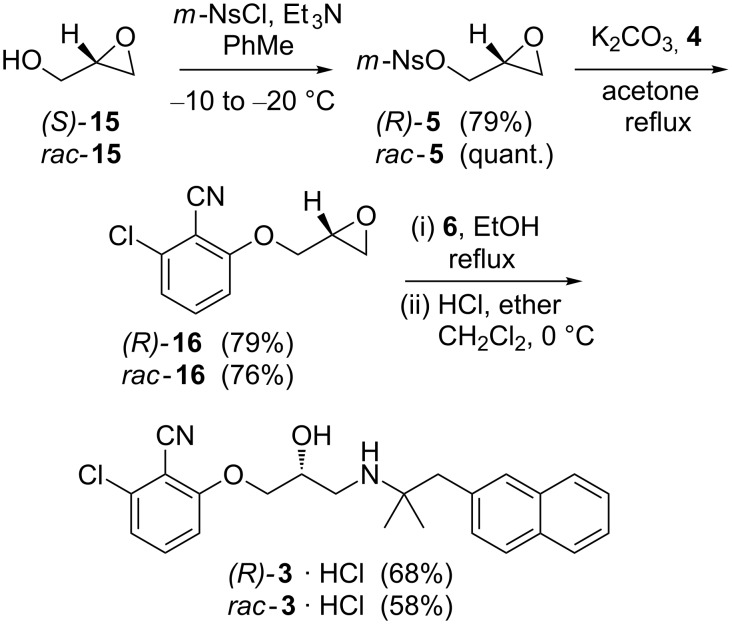
Synthesis of *rac*-**3** and (*R*)-**3** from commercially available racemic- and (*S*)*-*glycidol **15**, respectively. Only the optically active compounds are depicted.

With compounds **4** and **6** in hand we turned our attention to the synthesis of the remaining building block **5** (Scheme 4). In order to accurately determine the optical purity of the target molecule (*R*)-**3** by chiral HPLC, we performed the synthesis of racemic *rac*-**3** and optically pure (*R*)-**3** in parallel using the method by Marquis et al. [13]. Commercially available glycidol **15** activated as the *m*-nitrobenzene sulfonyl (*m*-nosyl) ester **5** has previously been shown to be an ideal leaving group in reactions with aryloxy nucleophiles (e.g., **4**) as it promotes direct S_N_2 over S_N_2’ attack thereby suppressing racemisation [15]. *m*-Nosylate **5** was synthesised by using nosyl chloride at low temperature [19]. Upon cooling, the reaction mixture became a viscous slurry, and it was important to maintain vigorous stirring to ensure full conversion. In this manner, a spectroscopically pure product could be obtained in quantitative yield after simple aqueous work-up (e.g., *rac-***5**). Alternatively, any residual alcohol **15** could be removed by chromatographic purification (e.g., (*R*)*-***5**). Next, the activated glycidols **5** were treated with phenol **4** in acetone under reflux and basic conditions to provide epoxides **16**. Epoxides **16** were obtained in good yield after purification by column chromatography and subsequent recrystallization to remove residual starting material **5**. Finally the target molecules *rac*-**3** and (*R*)-**3** were synthesised by ring-opening epoxides **16** with amine **6** in ethanol under reflux. ^1^H NMR analysis of the crude product showed the presence of approximately 5–10% of a structurally similar side-product that proved extremely difficult to remove by chromatography. The side product is likely the regioisomer formed by nucleophilic attack on the more sterically hindered epoxide carbon. Fortunately, the side product could be removed by recrystallization of the hydrochloric salt of **3** to produce **3**·HCl of excellent purity and in good overall yield.

**Figure 2:**
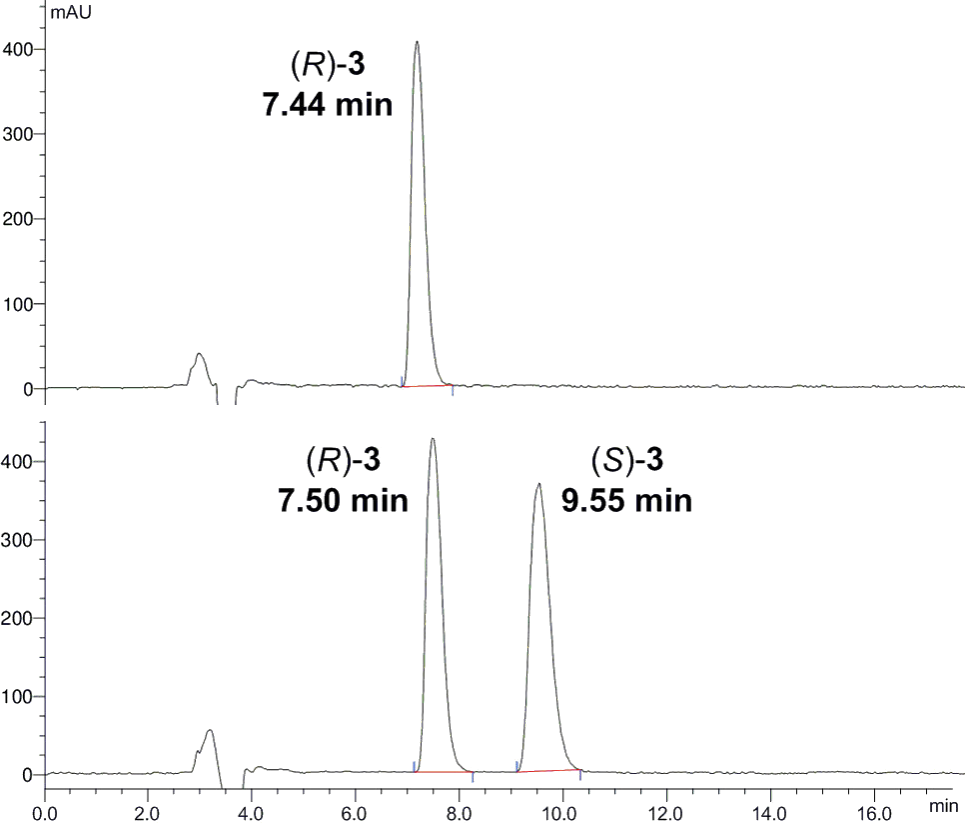
Determination of the optical purity for (*R*)-**3** by chiral HPLC on a Daicel AD-H column. Top: Optically enriched product (er > 99:1). Bottom: Racemic sample.

Analysis of optical purity by chiral HPLC showed that (*R*)-**3** was of excellent purity with an er > 99:1 (Figure 2).

**Figure 3:**
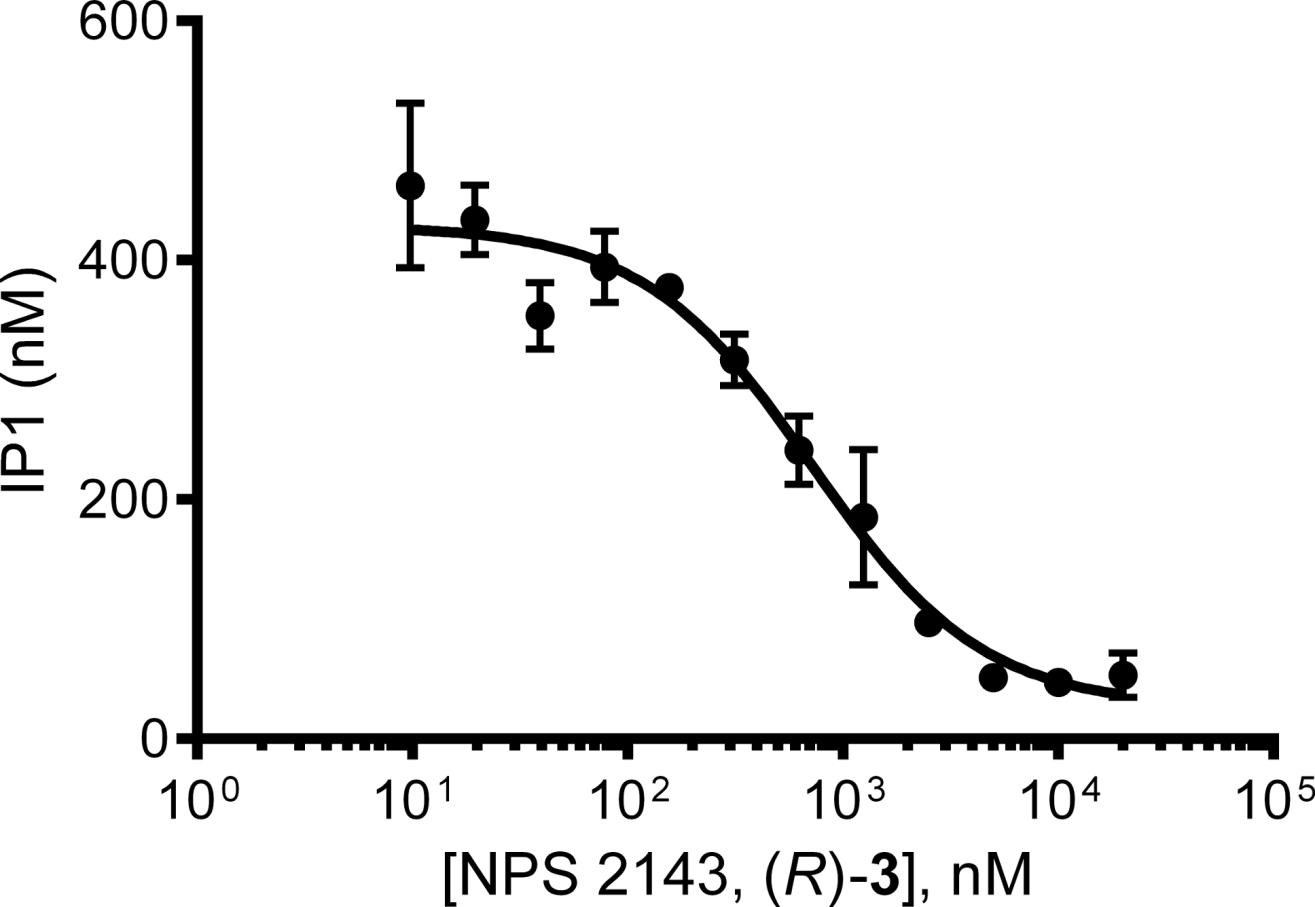
Characterisation of concentration-dependent (*R*)-**3** inhibition of 3.5 mM calcium-stimulated IP_1_ response in HEK293 cells stably transfected with rat CaSR. The graph is representative of three independent experiments.

Pharmacological testing of (*R*)-**3** was performed as previously described [20]. (*R*)-**3** showed inhibition of calcium-stimulated D-*myo*-inositol monophosphate (IP_1_) accumulation in a concentration-dependent manner, with a potency consistent with that reported in the literature [21–22] (IC_50_ = 0.64 μM, pIC_50_ = 6.3 ± 0.2), thus confirming its biological activity (Figure 3).

## Conclusion

Herein, for the first time, we disclose a detailed, reproducible and scalable synthetic procedure for the calcilytic ligand (*R*)-**3** (NPS 2143) making this important pharmacological tool compound readily available to the scientific community. All compounds have been characterised carefully and the optical purity of (*R*)-**3** was deemed excellent by chiral HPLC (er > 99:1). Moreover, the pharmacological profile of (*R*)-**3** was consistent with that reported in the literature.

## Supporting Information

^1^H and ^13^C NMR spectra and HPLC chromatograms for compounds **3**, **4**, **5**, **6**, **8**, and **16**. Chromatograms from chiral HPLC analysis of *rac*-**3** and (*R*)-**3** and experimental details for the pharmacological characterisation for (*R*)-**3**.

File 1 Experimental procedures and full characterisation of the calcilytic ligand NPS 2143.
