# Supplementary material for: Synthesis of the calcilytic ligand NPS 2143
Source: Beilstein J Org Chem. 2013 Jul 9;9:1383–7. doi: 10.3762/bjoc.9.154 (PMC3740522; doi:10.3762/bjoc.9.154)
Supplement: File 1 — Experimental procedures and full characterisation of the calcilytic ligand NPS 2143. [file Beilstein_J_Org_Chem-09-1383-s001.pdf]

Supporting Information

for

Synthesis of the calcilytic ligand NPS 2143

Henrik Johansson[1], Thomas Cailly[1,2], Alex Rojas Bie Thomsen[1], Hans Bräuner-Osborne[1] and Daniel Sejer Pedersen*[1]

Address: [1]Department of Drug Design and Pharmacology, University of Copenhagen, Universitetsparken 2, DK-2100 Copenhagen, Denmark and [2]Université de Caen Basse-Normandie, CERMN (EA 4258 - FR CNRS 3038 INC3M - SF 4206 ICORE) UFR des Sciences Pharmaceutiques, Bd Becquerel, F-14032 Caen, France.

Email: Daniel Sejer Pedersen - daniel.pedersen@sund.ku.dk

* Corresponding author

Experimental procedures and full characterisation of the calcilytic ligand NPS 2143.

Table of contents:

Experimental section S2

^{1}H, ^{13}C, and 2D NMR spectra S14

HPLC chromatograms S24

Determination of optical purity S27

Pharmacology S31

References S32

Experimental section

General information

All anhydrous reactions were carried out in oven or flame-dried glassware, under nitrogen. Solvents were of chromatography grade and dried using an SG Water solvent purification system (CH_2Cl_2, THF) or with 3 Å molecular sieves (Et_2O, MeCN, EtOH, DMSO, acetone and toluene). Commercially acquired chemicals were used without further purification. Aqueous sulfate buffer (pH ≈ 2) was prepared by dissolving 1.5 mol Na_2SO_4 in 0.5 mol H_2SO_4 and adding H_2O to a total volume of 2000 mL. Flash column chromatography and dry column vacuum chromatography (DCVC) [1] were carried out according to standard procedures using silica gel 60 (40–63 µm and 15–40 µm mesh, respectively).

Thin-layer chromatography (TLC)

TLC was carried out on Merck precoated silica gel 60 F_{254} plates and visualised using UV (254 nm), I_2/SiO_2, $KMnO_4$ or ninhydrin stain. Retention factor, R_f, values were rounded to the nearest 0.05.

Melting point (mp)

Melting points were recorded on a Stanford Research System (SRS) OptiMelt capillary melting-point apparatus and are uncorrected.

Fourier transform infrared spectroscopy (FTIR)

FT-IR was recorded neat on a Perkin-Elmer Spectrum One IR spectrometer with a universal ATR accessory, and the signals are reported in wavenumbers

(cm^{-1}). Solid samples were either loaded directly or dissolved in CH_2Cl_2 and loaded, then allowing the solvent to evaporate before recording the spectrum.

Optical rotation ($[\alpha]_D^{27}$)

Optical rotation values were measured at 27 °C on a Perkin-Elmer 241 polarimeter using a sodium vapor lamp (589 nm) and are reported in units of $10^{-1} \times deg \times dm^2 \times g^{-1}$. Anhydrous HPLC grade MeOH or $CHCl_3$ were used as solvent, and the concentration is reported as *c* (gram/100 mL).

Nuclear magnetic resonance spectroscopy (NMR)

NMR spectra were recorded on a 400 MHz Bruker Avance instrument. Signals are reported in ppm (δ), and solvents were used as the internal standard when assigning NMR spectra (δ ^1H NMR: $CDCl_3$ 7.26; DMSO-d_6 2.50. δ ^{13}C NMR: $CDCl_3$ 77.16; DMSO-d_6 39.52). Coupling constants (*J*) are given in hertz (Hz) and rounded to the nearest 0.5 Hz. Signal assignment was made from unambiguous chemical shifts and COSY, HSQC, HMBC and DEPTQ experiments.

High-performance liquid chromatography (HPLC)

HPLC-MS was recorded on an Agilent 1200 series system using an Xbridge RP C18 column, 3.5 µm, 100 × 4.6 mm, with UV detection at 210, 254 and 280 nm. Mobile phase (MP) A: 0.2% HCOOH, 99.8% H_2O (v/v). Mobile phase B: 0.2% HCOOH, 99.8% MeCN (v/v). Flow rate: 1 mL/min. Gradient: 0–7 min: 0–90% MP B, 7–9 min: 90% MP B, 9–16 min: 10% MP B.

Low-resolution mass spectrometry (LRMS)

LRMS were recorded on a Bruker Esquire 3000 plus instrument connected to an Agilent 1200 HPLC system, using an electrospray ionization (ESI) mass detector.

High-resolution mass spectrometry (HRMS)

High-resolution mass spectra were recorded on a Micromass Q-TOF 1.5, UB137.

Experimental

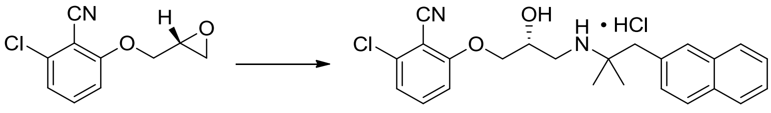

(*R*)-**16** (*R*)-**3**

***(R)*-2-Chloro-6-(2-hydroxy-3-((2-methyl-1-(naphthalen-2-yl)propan-2-yl)amino)propoxy)benzonitrile hydrochloride ((*R*)-3)**

According to the procedure by Marquis et al. [2] epoxide (*R*)-**16** (0.24 g, 1.13 mmol) and amine **6** (0.23 g, 1.13 mmol) were dissolved in anhydrous EtOH (6.5 mL) and stirred under reflux for 20 h. The mixture was allowed to cool to ambient temperature and then concentrated in vacuo to afford the crude product as a colorless foam. Purification by column chromatography (3% MeOH, 0.2% aqueous NH_3 in CH_2Cl_2, v/v) gave the free amine as a slightly yellow gum. The product was dissolved in CH_2Cl_2 (25 mL) and cooled to 0 °C using an ice-water bath. 2 M HCl in Et_2O (0.56 mL, 1.13 mmol) was added, and the solution was stirred for 10 min, then concentrated in vacuo to afford the crude product as an off-white solid. Recrystallization from EtOH/Et_2O afforded amine hydrochloride (*R*)-**3** (0.34 g, 68%) as white needles. **TLC** R_f = 0.25 (3% MeOH, 0.2% aqueous NH_3 in CH_2Cl_2, v/v); **HPLC**

t_R = 7.36 min; **IR** (neat) v_{max} = 3363, 2978, 2791, 2229, 1576; $[\alpha]_D^{27}$ = +21.3 (*c* = 1.0, MeOH); **^{1}H NMR** (400 MHz, DMSO-d_6): δ 9.23 (br t, *J* = 10.5 Hz, 1H, NH_AH$_B^+$), 8.86 (br t, *J* = 11 Hz, 1H, NH$_A$$H_B^+$), 7.92–7.88 (m, 3H, naphthyl-H4, H5, H8), 7.78 (s, 1H, naphthyl-H1), 7.70 (t, *J* = 8.5 Hz, 1H, Ar-H4), 7.54–7.48 (m, 2H, naphthyl-H6, H7), 7.39 (dd, *J* = 8.5, 1.5 Hz, 1H, naphthyl-H5), 7.34 (d, *J* = 8.5 Hz, 1H, Ar-H3), 7.31 (d, *J* = 8.5 Hz, 1H, Ar-H5), 6.06 (d, *J* = 4.5 Hz, 1H, OH), 4.37–4.27 (m, 3H, ArOCH_2C*H*), 3.41–3.36 (m, 1H, NCH_AH$_B$), 3.24–3.15 (m, 3H, NCH$_A$$H_B$, naphthyl-C$H_2$), 1.30 (s, 6H, 2 × CH_3); **^{13}C NMR** (100 MHz, DMSO-d_6): δ 161.6 (Ar-C6), 136.1 (Ar-C), 135.7 (Ar-C4), 132.9 (naphthyl-C), 132.8 (naphthyl-C), 131.9 (naphthyl-C2), 129.2 (naphthyl-C1), 129.0 (naphthyl-C3), 127.6 (2 × naphthyl-CH), 127.4 (naphthyl-C), 126.2 (naphthyl-CH), 125.9 (naphthyl-CH), 121.9 (Ar-C3), 113.6 (CN), 112.2 (Ar-C5), 101.8 (Ar-C1), 71.2 (Ar-OCH$_2$), 65.2 (HOCH), 59.7 (*C*(CH$_3$)$_2$), 43.8 (NCH$_2$), 42.7 (naphthyl-CH$_2$), 22.4 (CH_3), 22.3 (CH_3); **HRMS** *m*/*z* (ESI+) found: 431.1520 [M + Na]$^+$; $C_{24}H_{25}ClN_2NaO_2^+$ requires *M*, 431.1502.

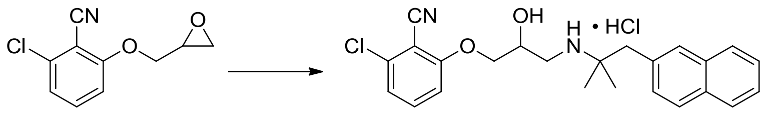

rac-**16** *rac*-**3**

2-Chloro-6-(2-hydroxy-3-((2-methyl-1-(naphthalen-2-yl)propan-2-yl)amino)propoxy)benzonitrile hydrochloride (*rac*-3)

Synthesized according to the procedure for (*R*)-**3** using epoxide *rac*-**16** (0.24 g, 1.13 mmol), amine **6** (0.23 g, 1.13 mmol) and anhydrous EtOH (6.5 mL). Column chromatography (2% MeOH, 0.2% aqueous NH_3 in CH_2Cl_2, v/v) gave the crude product as a slightly yellow gum. 2 M HCl in Et_2O (0.56 mL, 1.13 mmol) was added to give the hydrochloric salt.

Recrystallization from EtOH/Et_2O afforded amine hydrochloride *rac*-**3** (0.29 g, 58%) as white needles.

All analytical data for *rac*-**3** were identical to those reported above for (*R*)-**3**.

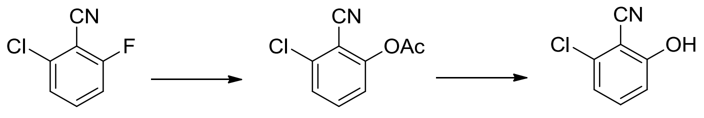

13 **14** **4**

2-Chloro-6-hydroxybenzonitrile (4)

Acetate formation: 2-Chloro-6-fluorobenzonitrile **13** (1.02 g, 6.58 mmol) was dissolved in anhydrous DMSO (25 mL). Potassium acetate (1.30 g, 13.2 mmol) was added and the mixture was stirred at 70 °C under nitrogen. Additional potassium acetate (0.32 g, 3.26 mmol) was added twice after 26 h and 30 h. After stirring for 54 h in total the reaction mixture was allowed to cool, EtOAc (60 mL) was added, and the mixture was transferred to a separation funnel. The organic phase was washed with water (2 × 40 mL) and brine (50 mL), and dried (Na_2SO_4). The combined aqueous phases were extracted with EtOAc (3 × 20 mL), and the organic phase was washed with brine (40 mL) and dried (Na_2SO_4). The two organic phases were pooled and concentrated in vacuo to afford the crude acetate **14** (1.02 g) as an off-white solid.

Phenol formation: The crude acetate **14** was dissolved in THF (35 mL) and 1 M aqueous LiOH (13.5 mL, 13.5 mmol) was added. The mixture was stirred at ambient temperature for 4.5 h and the solvent was removed under reduced pressure. Water (100 mL) was added and the basic (pH = 13–14) aqueous phase was washed with EtOAc (4 × 30 mL). 4 M aqueous HCl was added to the aqueous phase until pH = 3, and the aqueous phase was extracted with

EtOAc (3 × 40 mL). The organic phase was washed with brine (50 mL), dried (Na_2SO_4) and concentrated in vacuo to give phenol **4** (0.60 g, 59% over two steps) as an off-white solid, which was used without further purification. **TLC** R_f = 0.25 (40% EtOAc in *n*-heptane, v/v); **HPLC** t_R = 7.58 min; **IR** (neat) v_{max} = 3276, 2243, 1597; **^{1}H NMR** (400 MHz, DMSO-d_6): δ 11.63 (s, 1H, OH), 7.49 (dd, *J* = 8.5, 8 Hz, 1H, Ar-H4), 7.09 (dd, *J* = 8, 1 Hz, 1H, Ar-H5), 6.99 (dd, *J* = 8.5, 1 Hz, 1H, Ar-H3); **^{13}C NMR** (100 MHz, DMSO-d_6): δ 162.0 (Ar-C6), 135.6 (Ar-C2), 135.2 (Ar-C4), 120.0 (Ar-C5), 114.9 (Ar-C3), 114.1 (CN), 100.0 (Ar-C1); **HRMS** *m*/*z* (ESI-) found: 151.9783 [M − H]$^-$. $C_7H_3ClNO^-$ requires *M,* 151.9909.

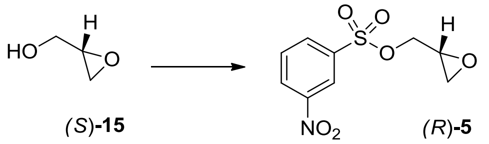

***(R)*-Oxiran-2-yl-methyl 3-nitrobenzenesulfonate ((*R*)-5)**

According to the procedure by Fagerström et al. [3] 3-nitrobenzene-1-sulfonyl chloride (3.15 g, 14.2 mmol) was dissolved/dispersed in anhydrous toluene (60 mL) and cooled in a dry ice/acetone bath at −10 °C to −20 °C. Triethylamine (2.1 mL, 15.1 mmol) and (*S*)-glycidol **15** (0.90 mL, 13.5 mmol) were added to give a slightly yellow slurry and the mixture was stirred for 20 h. The mixture was allowed to warm to room temperature and was transferred to a separation funnel with EtOAc (100 mL) and sulfate buffer (60 mL). The phases were separated and the organic phase was washed with sulfate buffer (60 mL), saturated $NaHCO_3$ (60 mL) and brine (60 mL), dried (Na_2SO_4), filtered and concentrated in vacuo to give the crude product as slightly yellow solid. Purification by DCVC [id 6.5 cm; 40 mL fractions; 5%

increments; 1 × 0–25% and 2 × 30–100% EtOAc in *n*-heptane, v/v; the column was deactivated prior to sample loading by washing with 200 mL of 5% Et_3N in *n*-heptane, v/v, followed by 3 × 50 mL *n*-heptane] gave epoxide *(R)*-**5** (2.76 g, 79%) as an off-white solid. **TLC** R_f = 0.25 (40% EtOAc in *n*-heptane, v/v); **HPLC** t_R = 7.90 min; **IR** (neat) v_{max} = 3092, 1532, 1351; $[\alpha]_D^{27}$ = −19.7 (*c* = 1.0, MeOH); **^{1}H NMR** (400 MHz, $CDCl_3$): δ 8.78 (t, *J* = 2 Hz, 1H, Ar-H2), 8.53 (ddd, *J* = 8, 2, 1 Hz, 1H, Ar-H), 8.26 (ddd, *J* = 8, 2, 1 Hz, 1H, Ar-H), 7.81 (t, *J* = 8 Hz, 1H, Ar-H5), 4.48 (dd, *J* = 11.5, 3 Hz, 1H SO-CH_AH_B), 4.05 (dd, *J* = 11.5, 6.5 Hz, 1H, SO-CH_AH_B), 3.22 (ddt, *J* = 6.5, 4, 3 Hz, 1H, oxiran-CH), 2.85 (dd, *J* = 5, 4 Hz, 1H, oxiran-$CH_{syn}H_{anti}$), 2.63 (dd, *J* = 5, 3 Hz, 1H, oxiran-$CH_{syn}H_{anti}$); **^{13}C NMR** (100 MHz, $CDCl_3$): δ 148.4 (Ar-C3), 138.3 (Ar-C1), 133.5 (Ar-C2), 130.9 (Ar-C), 128.6 (Ar-C), 123.4 (Ar-C5), 71.8 (SO-CH_2), 48.8 (oxiran-CH), 44.7 (oxiran-CH_2); **HRMS** *m*/*z* (ESI+) found: 282.0044 $[M + Na]^+$; $C_9H_9NNaO_6S$ requires *M,* 282.0043.

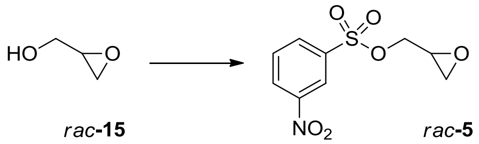

rac-**15** *rac*-**5**

Oxiran-2-ylmethyl 3-nitrobenzenesulfonate (*rac*-5)

Synthesized as described for *(R)*-**5** using 3-nitrobenzene-1-sulfonyl chloride (3.15 g, 14.2 mmol), toluene (60 mL), triethylamine (2.1 mL, 15.1 mmol) and racemic glycidol **15** (0.90 mL, 13.5 mmol) for 24 h. The crude epoxide *rac*-**5** (3.51 g, quantitative) was isolated as an off-white solid and used without further purification.

All analytical data for *rac*-**5** were identical to those reported above for (*R*)-**5**.

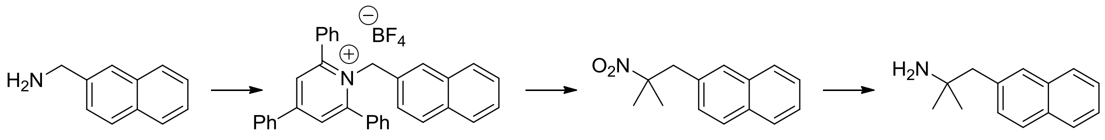

8 10 12 6

2-Methyl-1-(naphthalen-2-yl)-propan-2-amine (6)

By a method similar to that of Katritzky and co-workers [4] 2,4,6-triphenylpyrylium tetrafluoroborate **9** (5.51 g, 13.9 mmol) and naphthalen-2-ylmethanamine **8** (2.41 g, 29.0 mmol) were suspended in absolute ethanol (50 mL) and the red suspension was stirred vigorously overnight under nitrogen. Et_2O (100 mL) was added and a light brown precipitate was collected by filtration, washed with Et_2O (4 × 25 mL) and dried in a vacuum desiccator over potassium hydroxide. The pyridinium salt **10** (5.17 g, 63%) was obtained as a light brown amorphous solid that was used without further purification.

Sodium methoxide (0.76 g, 14.0 mmol) was suspended in anhydrous toluene (10 mL) and 2-nitropropane (1.26 mL, 14.0 mmol) was added dropwise with vigorous stirring. After 20 min the suspension was concentrated in vacuo to give a white solid. Anhydrous DMSO (20 mL) was added followed by *N*-(4-methylbenzyl)-2,4,6-triphenylpyridinium tetrafluoroborate **10** (2.50 g, 4.7 mmol) and the resulting brown slurry was stirred vigorously overnight at 60 °C under nitrogen. After stirring for 13 h water (50 mL) was added, and the mixture was transferred to a separatory funnel and extracted with Et_2O (100 + 2 × 50 mL). The combined organic phases were washed with brine (100 mL). Amberlyst 15 strong acid resin (34 g) was added to the organic phase and stirred vigourously. After 1 h the resin was removed by filtration and washed with Et_2O (200 mL). The combined organic phases were concentrated in

vacuo to give crude 2-(2-methyl-2-nitropropyl)naphthalene **12** (1.2 g) as a yellow amorphous solid that was used without further purification.

The crude nitro compound **12** (1.2 g) was suspended in absolute ethanol (50 mL) and zinc dust (particle size <10 μm, 6.3 g, 95.0 mmol) was added. The slurry was cooled in an ice/water bath and 4 M aqueous HCl (25 mL) was added dropwise. After 15 min the cooling bath was removed and the mixture was stirred vigorously overnight. The reaction mixture was transferred to a 1 L beaker with ethanol (70 mL) and cooled in an ice-water bath. Saturated aqueous $NaHCO_3$ was added slowly to pH 8 (≈100 mL) and the cooling bath was removed. The slurry was filtered on filter paper and washed with ethanol (100 mL). The organic phases were combined and most of the ethanol was removed in vacuo. The remaining solution was transferred to a separatory funnel and extracted EtOAc (200 + 2 × 100 mL). The combined organic phases were washed with 1:1 brine/saturated aqueous $NaHCO_3$ (200 mL), dried (Na_2SO_4), filtered and concentrated in vacuo to give a yellow oil that crystallised to give needles upon standing. Purification on a short DCVC column [id. 4 cm × h. 2 cm; 20 mL fractions; 2 × heptanes; 4 × 50% EtOAc in heptanes (v/v); 5–50% MeOH, 0.5% Et_3N in EtOAc (v/v)] gave amine **6** (0.69 g, 73% over two steps) as tan needles. **TLC** R_f = 0.2 (5% MeOH, 0.4% aqueous NH_3 in CH_2Cl_2, v/v); **HPLC** t_R = 6.15 min; **IR** (neat) ν_{max} = 3356, 2960, 1598; **^{1}H NMR** (400 MHz, $CDCl_3$): δ 7.87–7.84 (m, 2H, naphthyl-H5, H8), 7.81 (d, J = 8.5 Hz, 1H, naphthyl-H4), 7.68 (br s, 1H, naphthyl-H1), 7.49–7.42 (m, 2H, naphthyl-H6, H7), 7.39 (dd, J = 8.5, 2 Hz, 1H, naphthyl-H3), 2.74 (s, 2H; CH_2), 1.44 (br s, 2H; NH_2), 1.03 (s, 6H, 2 × CH_3); **^{13}C NMR** (100 MHz, DMSO-d_6): δ 136.7 (naphthyl-C8a), 132.8 (naphthyl-C4a), 131.6 (naphthyl-C2), 129.4 (naphthyl-C3), 128.3 (naphthyl-C1), 127.4 (naphthyl-C5 and C8),

126.8 (naphthyl-C4), 125.7 (naphthyl-C6 or C7), 125.1 (naphthyl-C6 or C7), 50.5 (CH_2), 50.1 (CNH_2), 30.2 (CH_3); **HRMS** *m*/*z* (ESI+) found: 200.1438 $[M + H]^+$; $C_{14}H_{18}N^+$ requires *M,* 200,1434.

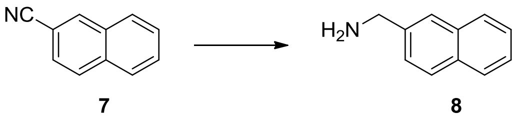

Naphthalen-2-ylmethanamine (8)

A 2 L three-neck round-bottom flask fitted with a reflux condenser, a pressure equalizing addition funnel and a nitrogen gas bubbler was charged with solid $LiAlH_4$ (6.19 g, 163.2 mmol) and cooled in an ice-water bath. Anhydrous Et_2O (400 mL) was added under stirring followed by dropwise addition over 1 h of 2-cyanonaphthalene **7** (10.1 g, 65.3 mmol) dissolved in anhydrous Et_2O (250 mL). The reaction mixture was stirred vigorously at room temperature for 4 h and then cooled with acetone/dry ice to −78 °C. EtOAc (200 mL) was added dropwise over 30 min and the cooling bath was removed. Half saturated aqueous sodium, potassium tartrate (300 mL) was added over 30 min, and the resultant slurry stirred was vigorously. The phases were separated, and the aqueous phase was extracted with EtOAc (200 mL). Brine (100 mL) was added to facilitate the separation of the phases. The combined organic phases were dried (Na_2SO_4), filtered and concentrated in vacuo to give a yellow amorphous solid. The crude product was dissolved in ethyl acetate (300 mL) under reflux, and the hot solution was extracted with 1 M aqueous HCl (3 × 100 mL). The pH of the combined aqueous phases was adjusted to 12 by addition of 35% aqueous NaOH and extracted with EtOAc (3 × 100 mL). The organic phases were washed with brine (100 mL), dried (Na_2SO_4), filtered and concentrated in vacuo to give amine **8** (10.1 g, 98%) as

a tan amorphous solid that was used without further purification. **TLC** R_f = 0.2 (5% MeOH, 0.4% aqueous NH_3 in CH_2Cl_2, v/v); **HPLC** t_R = 5.51 min; **IR** (neat) v_{max} = 3281, 3054, 1598, 1561; **^{1}H NMR** (400 MHz, DMSO-d_6): δ 7.88–7.81 (m, 4H), 7.52–7.43 (m, 3H) (7 × naphthyl-H), 3.89 (s, 2H; CH_2), 1.84 (s, 2H; NH_2); **^{13}C NMR** (100 MHz, DMSO-d_6): δ 141.5 (quaternary C), 133.0 (quaternary C), 131.9 (quaternary C), 127.5 (CH), 127.4 (CH), 127.4 (CH), 126.2 (CH), 125.9 (CH), 125.2 (CH), 124.7 (CH), 45.6 (CH_2); **HRMS** *m/z* (ESI+) found: 158.0969 $[M + H]^+$; $C_{11}H_{12}N^+$ requires *M,* 158.0964.

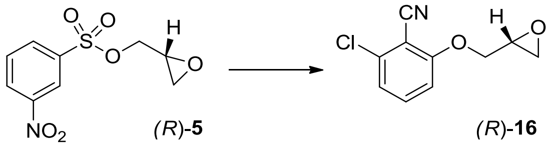

***(R)*-2-Chloro-6-(oxiran-2-ylmethoxy)benzonitrile ((*R*)-16)**

According to the procedure by Marquis et al. [2] phenol **4** (0.70 g, 4.56 mmol) was dissolved in dry acetone (45 mL). Potassium carbonate (1.89 g, 13.7 mmol) was added and the mixture was stirred under reflux for 45 min. The mixture was allowed to cool to ambient temperature and epoxide *(R)*-**5** (1.24 g, 4.79 mmol) was added. The resulting mixture was stirred under reflux for 20 h. After cooling to ambient temperature the solids were removed by filtration on a plug of celite and washed with acetone. The combined organic phases were concentrated in vacuo to give the crude product as a yellow solid. Purification by column chromatography (45% EtOAc in *n*-heptane, v/v) followed by recrystallization from EtOAc/*n*-heptane gave epoxide (*R*)-**16** (0.75 g, 79%) as white needles (three crops). **TLC** R_f = 0.4 (50% EtOAc in *n*-heptane, v/v); **mp** 127–127.5 °C (EtOAc/*n*-heptane); **HPLC** t_R = 8.31 min; **IR** (neat) v_{max} = 2231, 1589; $[\alpha]_D^{27}$ = +1.7 (*c* = 1.0, $CHCl_3$); **^{1}H NMR** (400 MHz,

$CDCl_3$): δ 7.44 (dd, J = 8.5 Hz, 1H, Ar-H4), 7.10 (dd, J = 8, 1 Hz, 1H; Ar-H5), 6.94 (dd, J = 8.5, 1 Hz, 1H, Ar-H3), 4.40 (dd, J = 11.5, 3 Hz, 1H, Ar-OC$H_A H_B$), 4.12 (dd, J = 11.5, 5.5 Hz, 1H, Ar-OC$H_A H_B$), 3.40 (ddt, J = 5.5, 4.5, 3 Hz, 1H, oxiran-CH), 2.94 (dd, J = 5, 4.5 Hz, 1H, oxiran-C$H_{syn}H_{anti}$), 2.85 (dd, J = 5, 2.5 Hz, 1H, oxiran-C$H_{syn}H_{anti}$); **^{13}C NMR** (100 MHz, $CDCl_3$): δ 161.6 (Ar-C6), 138.3 (Ar-C2), 134.4 (Ar-C4), 122.4 (Ar-C5), 113.5 (CN), 110.9 (Ar-C3), 103.9 (Ar-C1), 70.0 (Ar-OCH_2), 49.8 (oxiran-CH), 44.6 (oxiran-CH_2); **HRMS** *m*/*z* (ESI+) found: 232.0136 $[M + Na]^+$; $C_{10}H_8ClNNaO_2$ requires *M,* 232.0136.

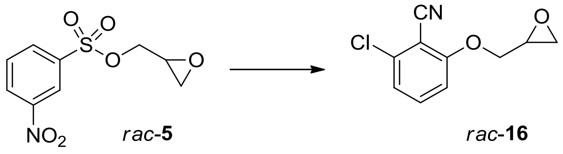

rac-**5** *rac*-**16**

2-Chloro-6-(oxiran-2-ylmethoxy)benzonitrile (*rac*-16)

Synthesised as described for (*R*)-**16** using phenol **4** (0.70 g, 4.56 mmol), potassium carbonate (1.89 g, 13.7 mmol) and epoxide *rac*-**5** (1.24 g, 4.79 mmol). Purification by column chromatography (45% EtOAc in *n*-heptane, v/v) followed by recrystallization from EtOAc/*n*-heptane gave epoxide *rac*-**16** (0.72 g, 76%) as white needles. **mp:** 102–103 °C (EtOAc/*n*-heptane).

All analytical data for *rac*-**16** were identical to those reported above for *(R)*-**16**.

NMR spectra

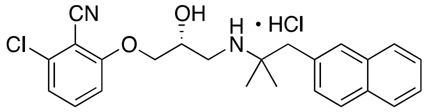

(*R*)-**3**

^{1}H NMR, 400 MHz, DMSO-*d*$_6$

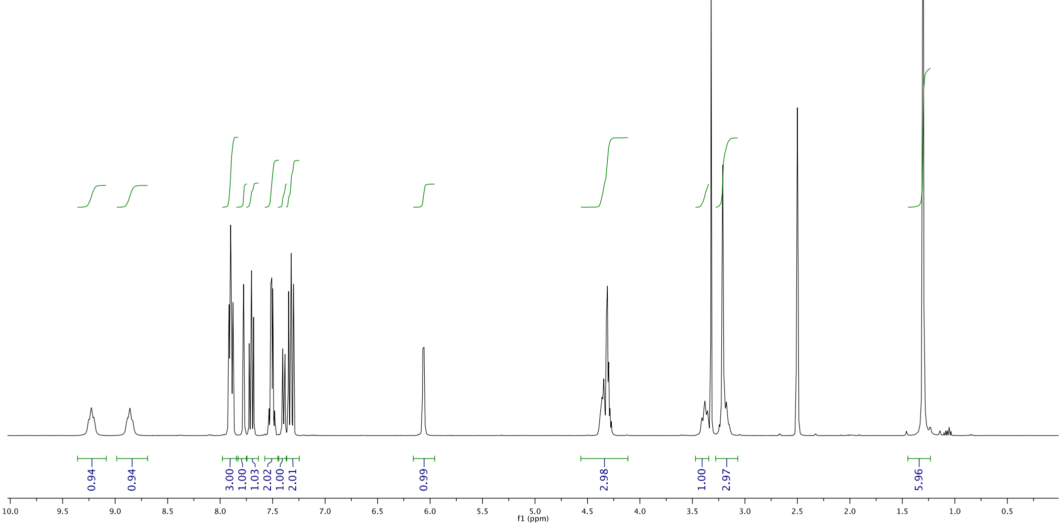

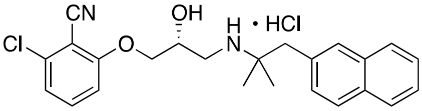

(*R*)-**3**

^{13}C NMR, 100 MHz, DMSO-*d*$_6$

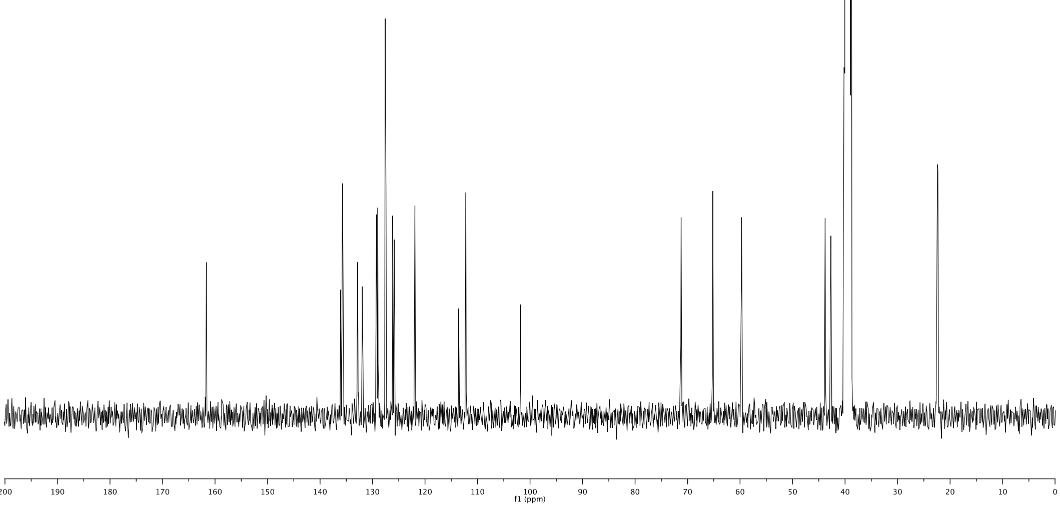

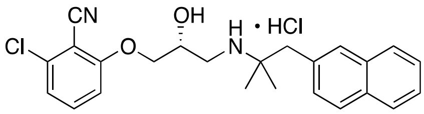

(*R*)-**3**

HSQC, DMSO-d_6

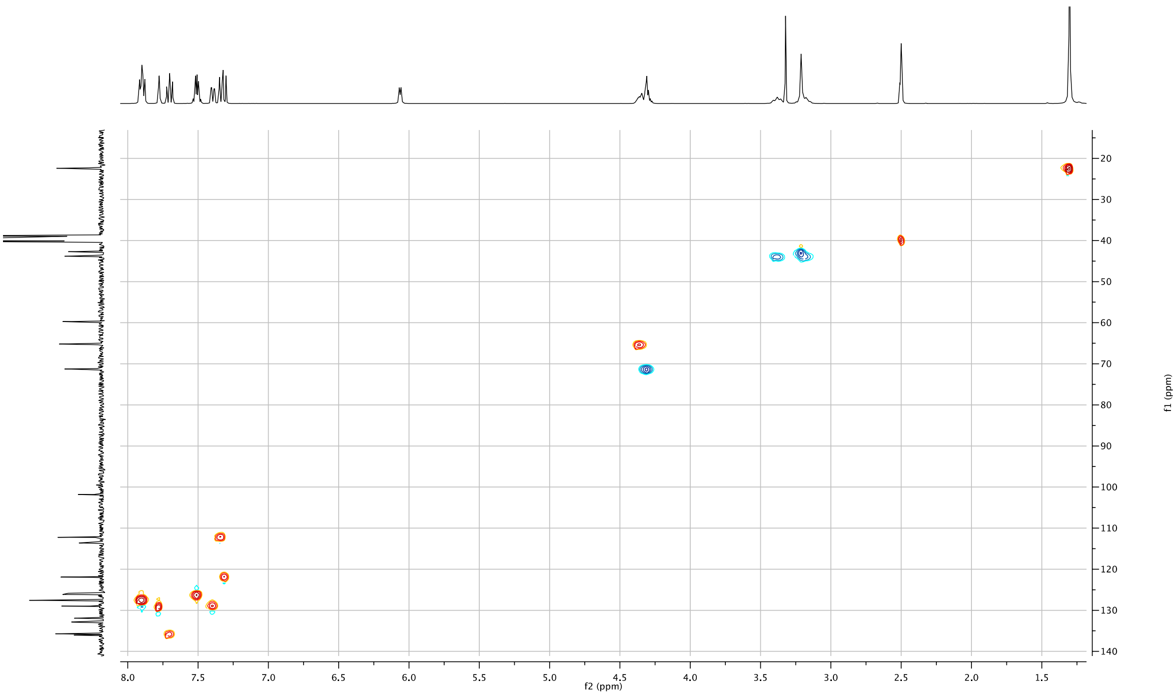

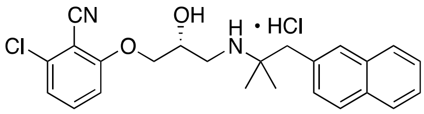

(*R*)-**3**

HMBC, DMSO-d_6

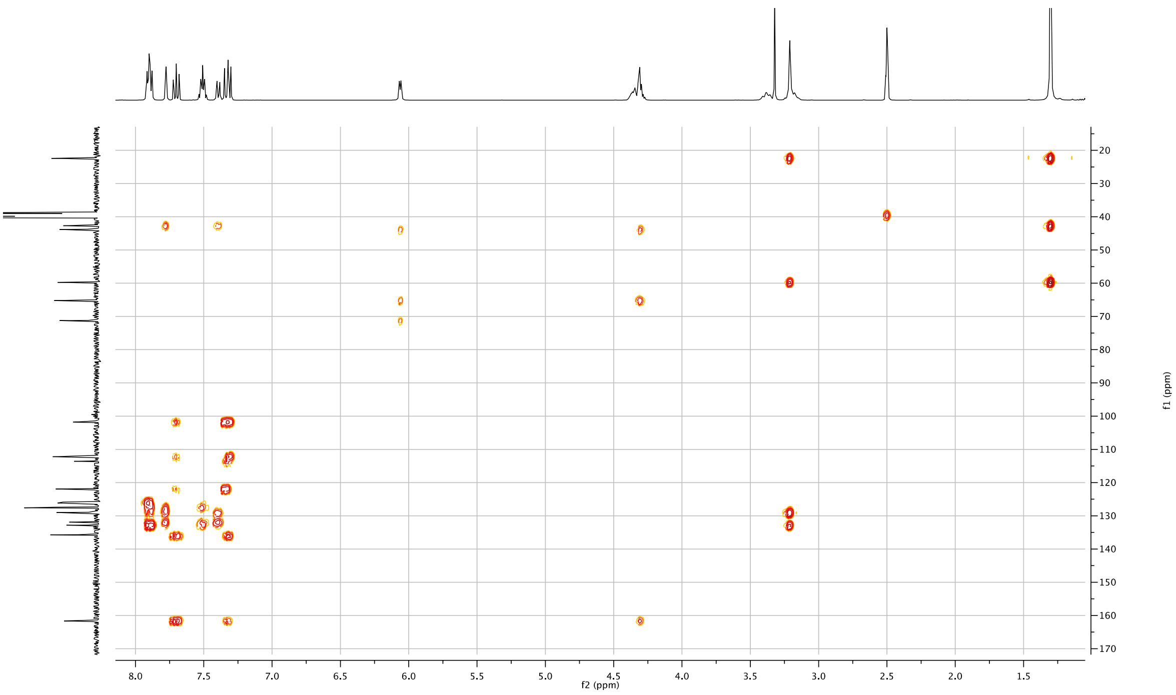

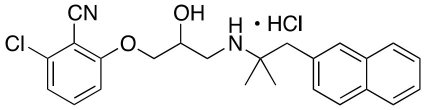

rac-**3**

^{1}H NMR, 400 MHz, DMSO-*d*$_6$

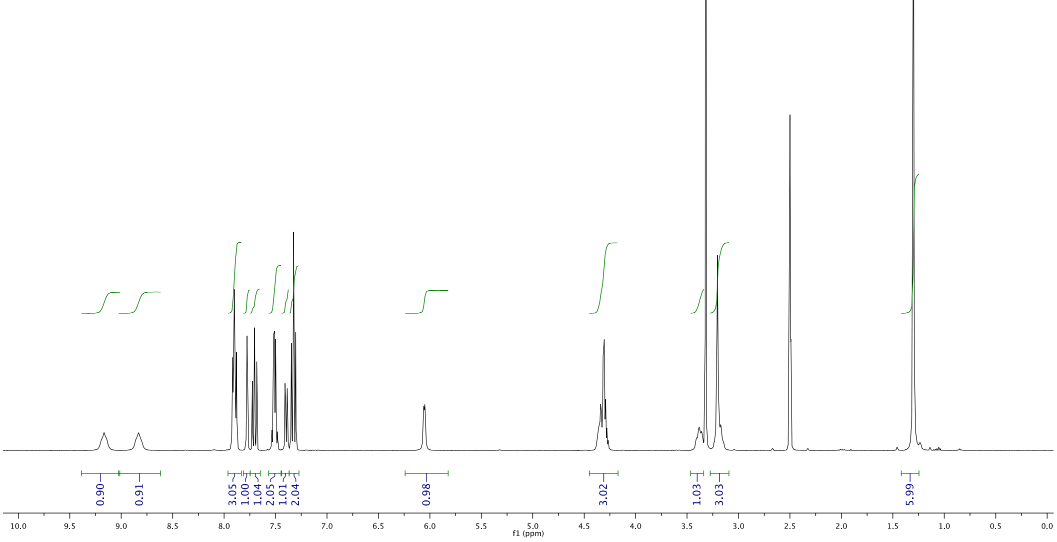

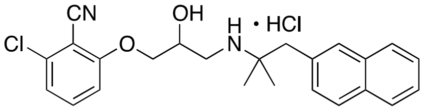

rac-**3**

^{13}C NMR, 100 MHz, DMSO-*d*$_6$

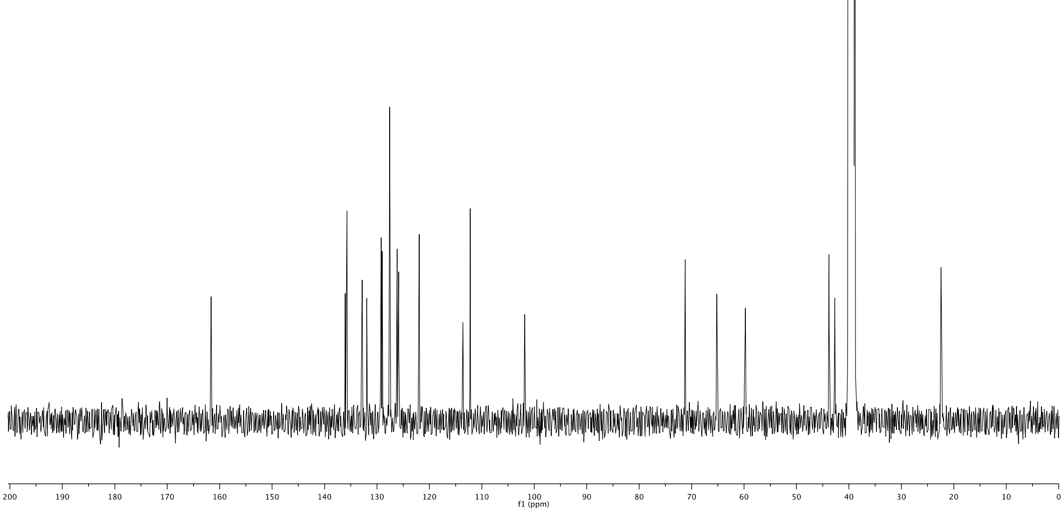

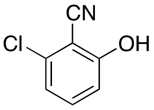

4

^{1}H NMR, 400 MHz, DMSO-d_6

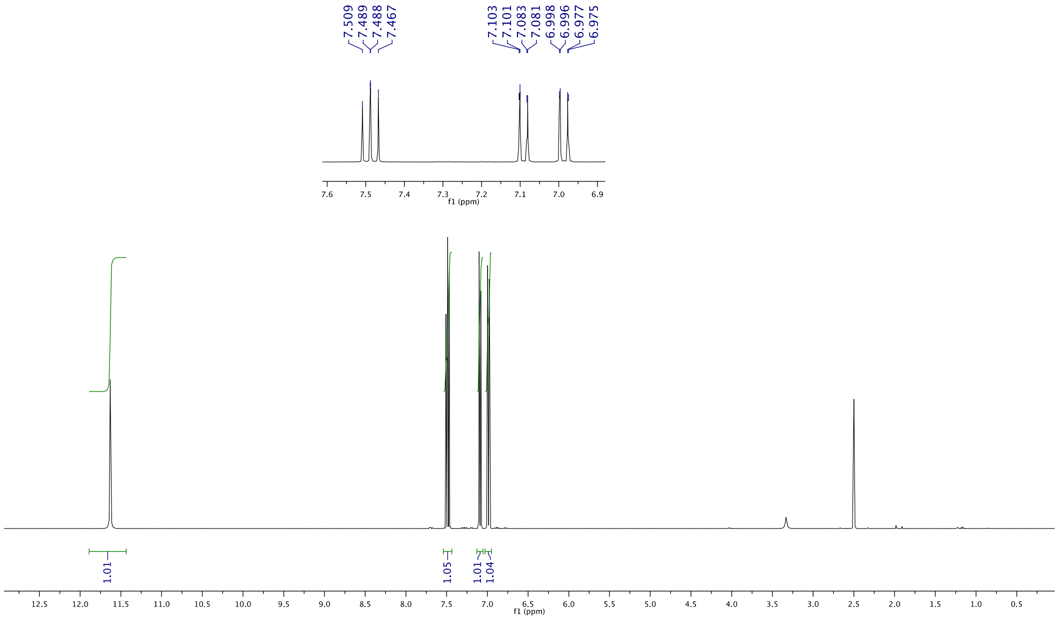

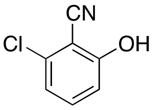

4

^{13}C NMR, 100 MHz, DMSO-d_6

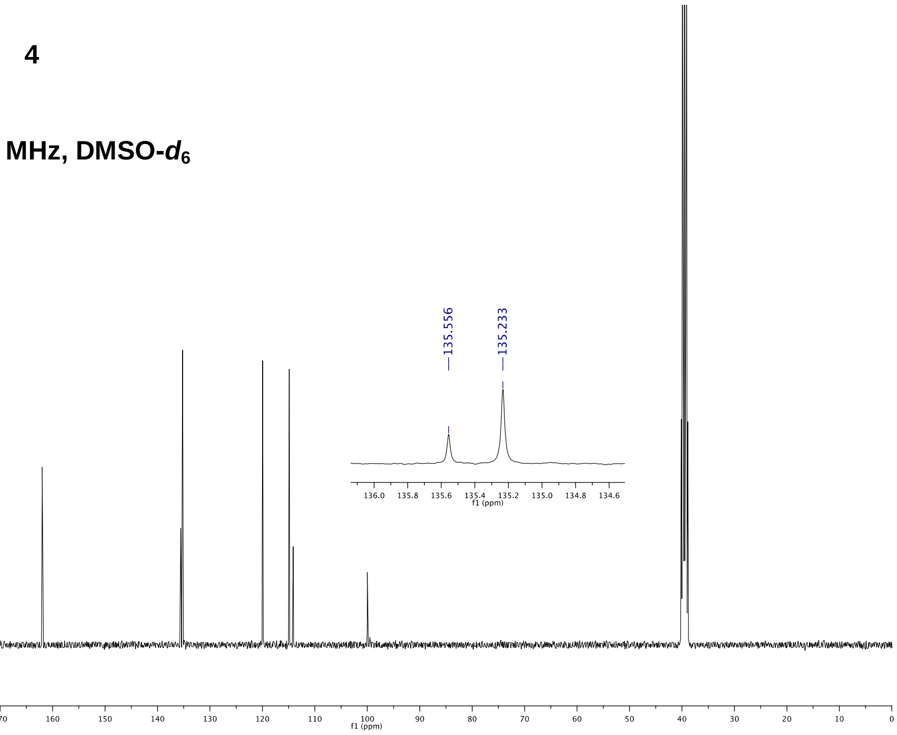

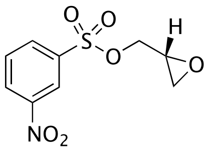

(*R*)-**5**

^{1}H NMR, 400 MHz, $CDCl_3$

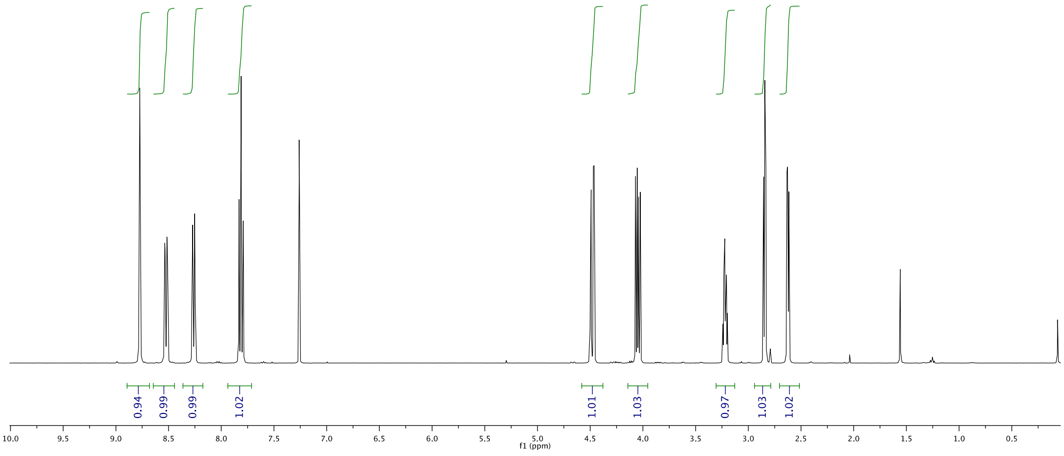

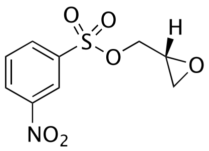

(*R*)-**5**

^{13}C NMR, 100 MHz, $CDCl_3$

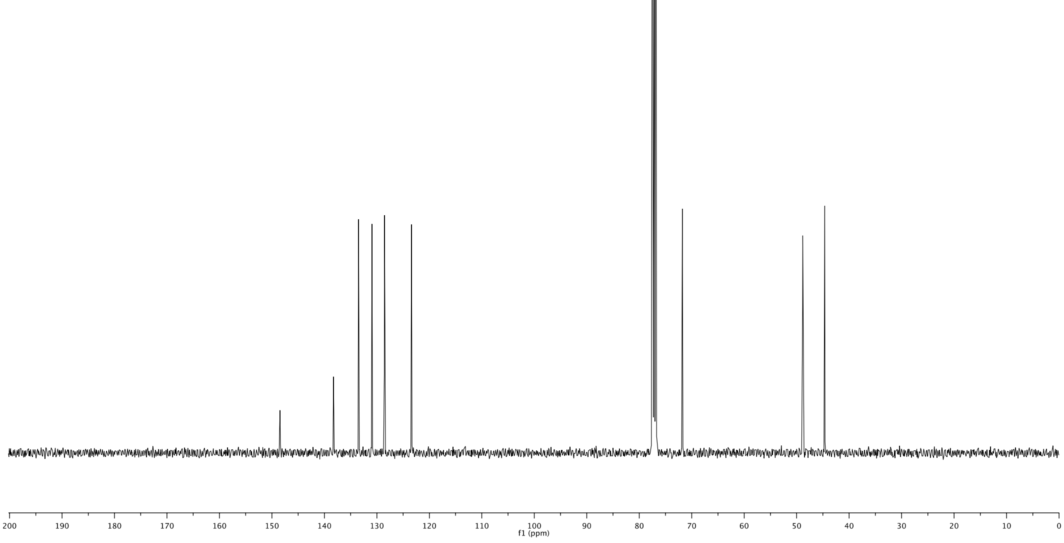

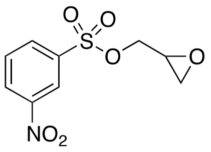

rac-**5**

^{1}H NMR, 400 MHz, $CDCl_3$

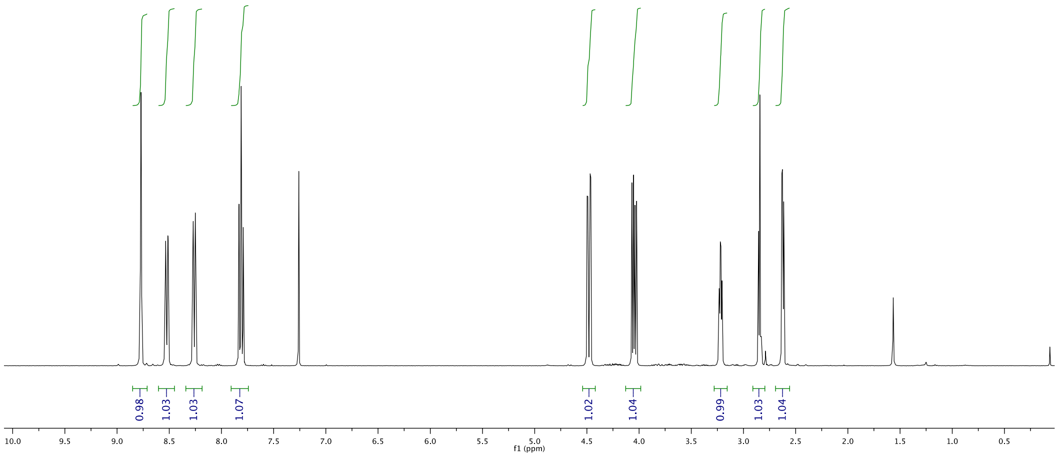

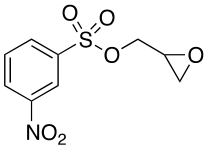

rac-**5**

^{13}C NMR, 100 MHz, $CDCl_3$

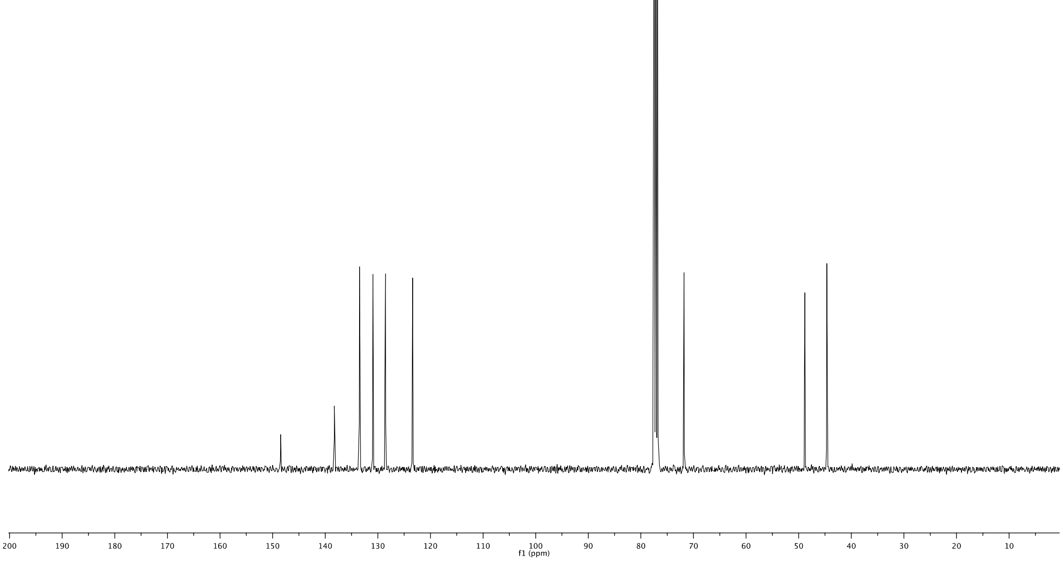

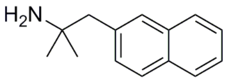

6

^{1}H NMR, 400 MHz, DMSO-*d*$_6$

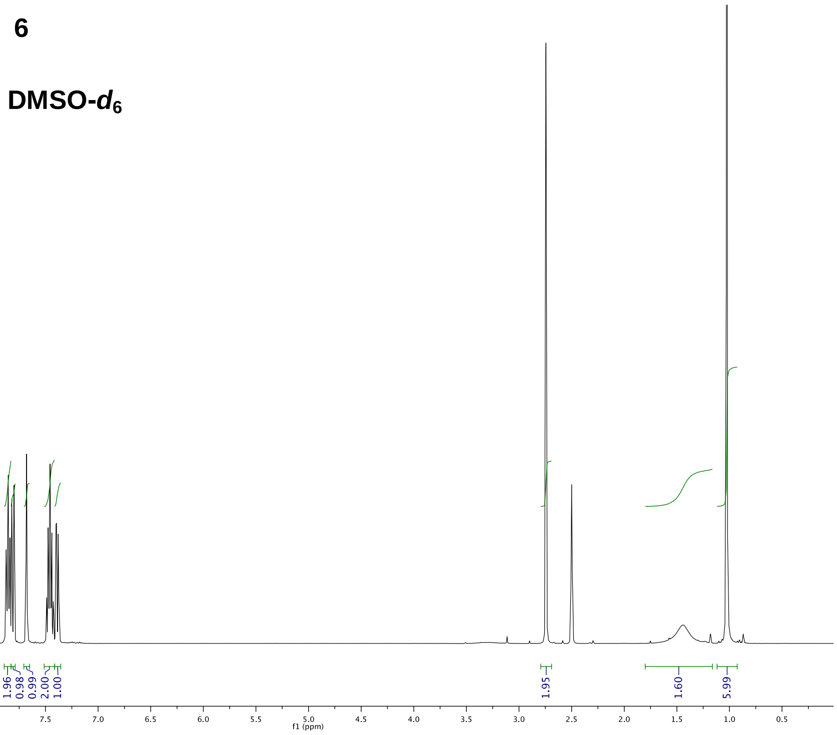

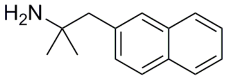

6

^{13}C NMR, 100 MHz, DMSO-*d*$_6$

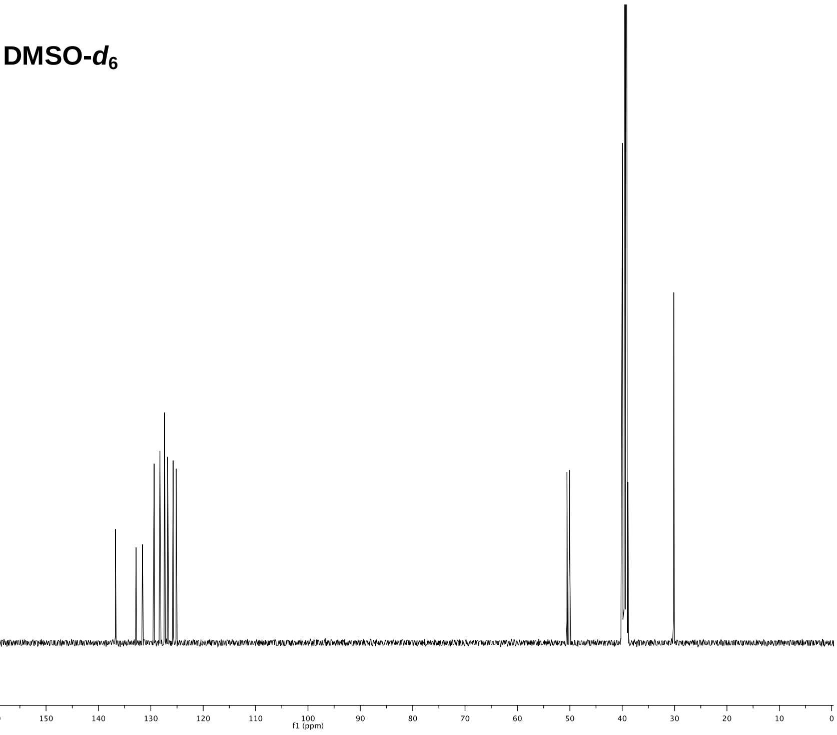

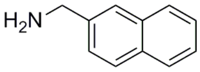

8

^{1}H NMR, 400 MHz, DMSO-*d_6*

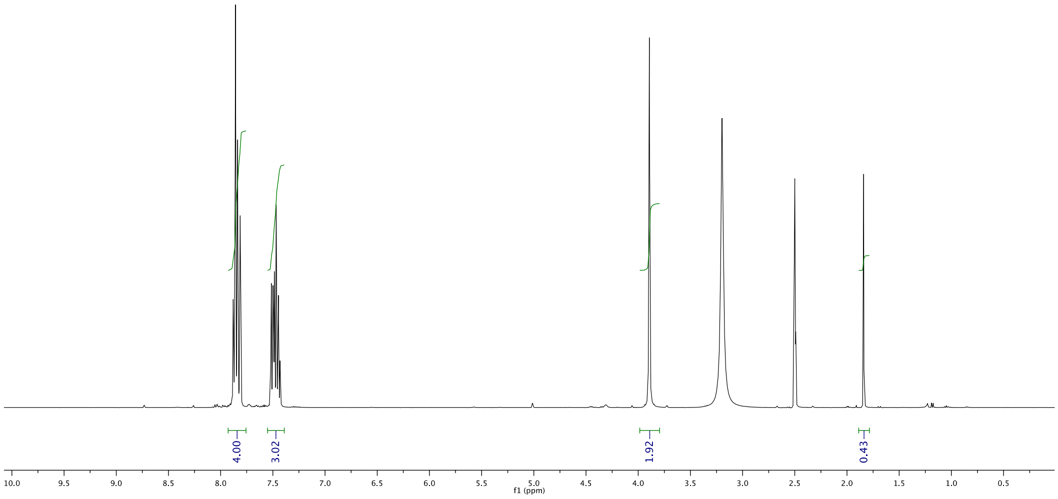

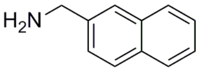

8

^{13}C NMR, 100 MHz, DMSO-*d_6*

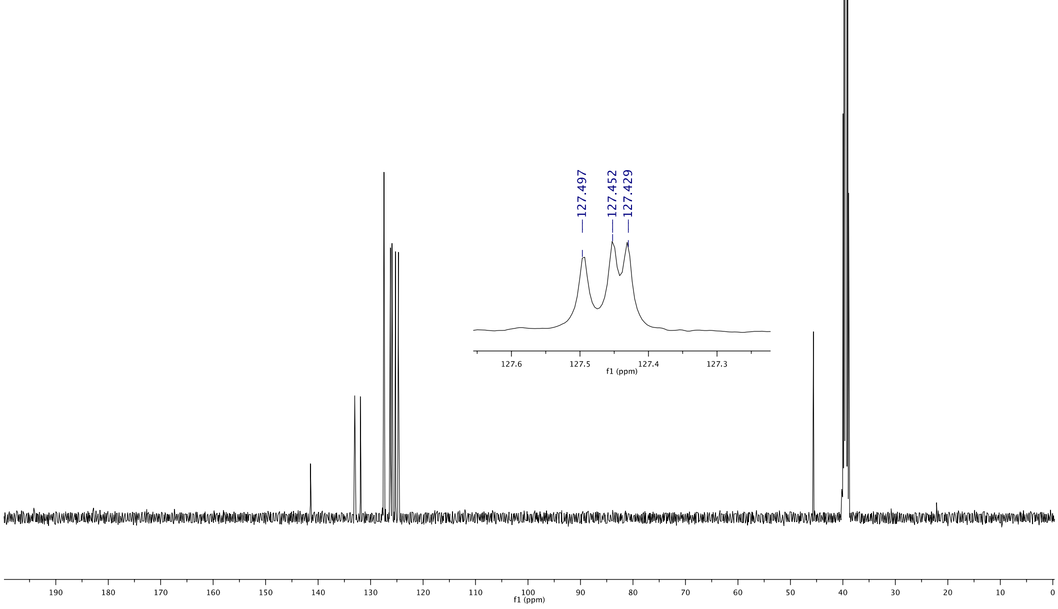

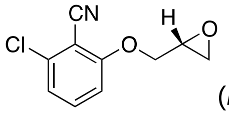

(*R*)-**16**

^{1}H NMR, 400 MHz, CDCl$_3$

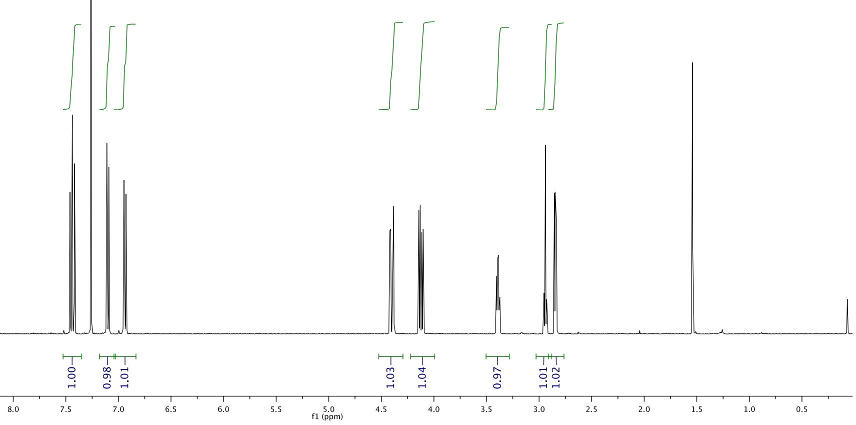

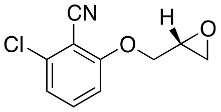

(*R*)-**16**

^{13}C NMR, 100 MHz, CDCl$_3$

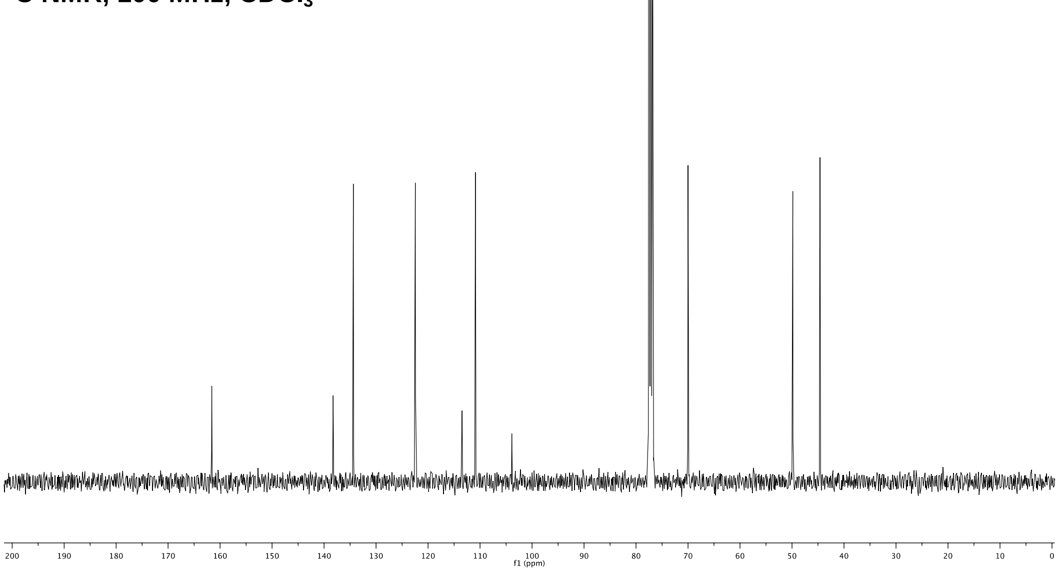

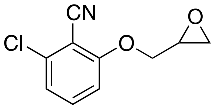

rac-**16**

^{1}H NMR, 400 MHz, $CDCl_3$

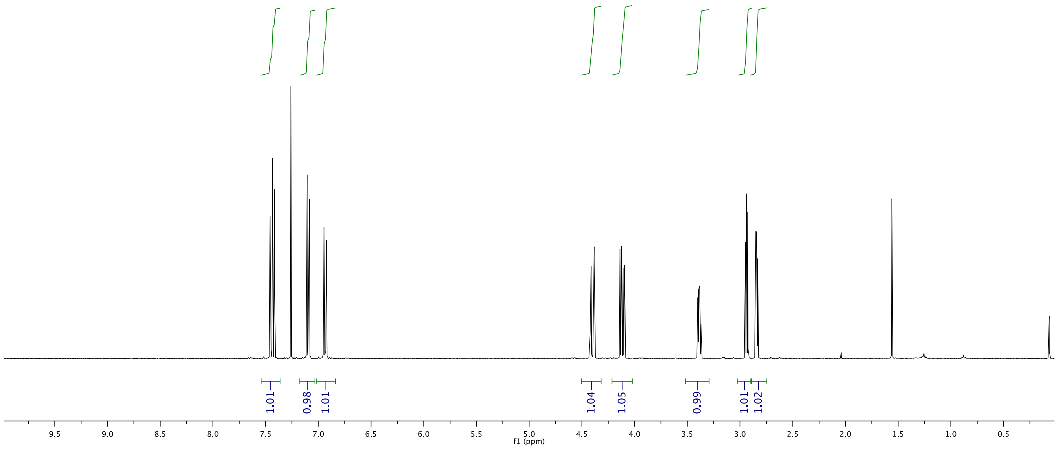

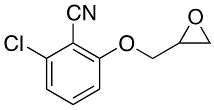

rac-**16**

^{13}C NMR, 100 MHz, $CDCl_3$

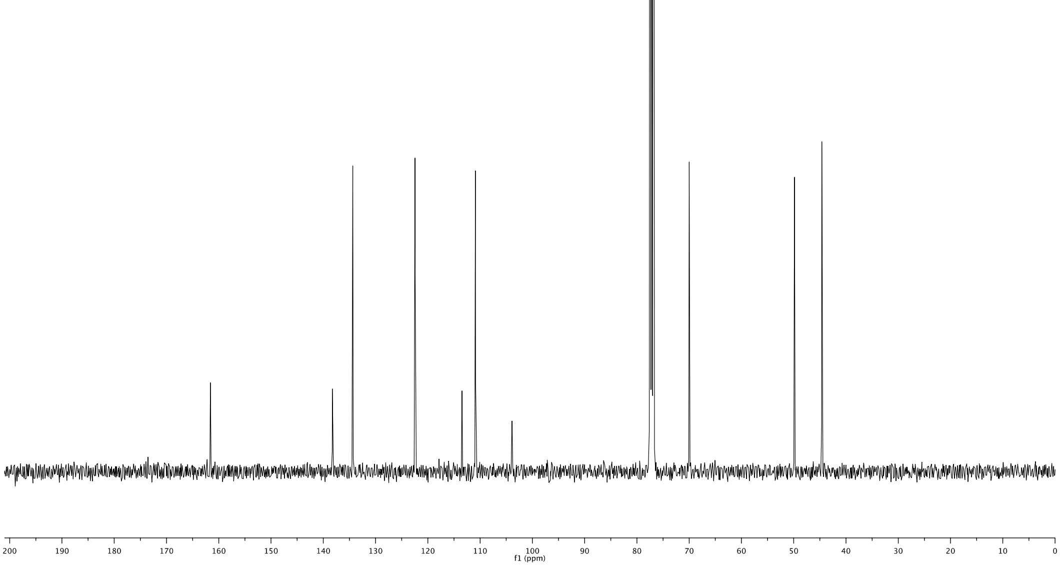

HPLC chromatograms

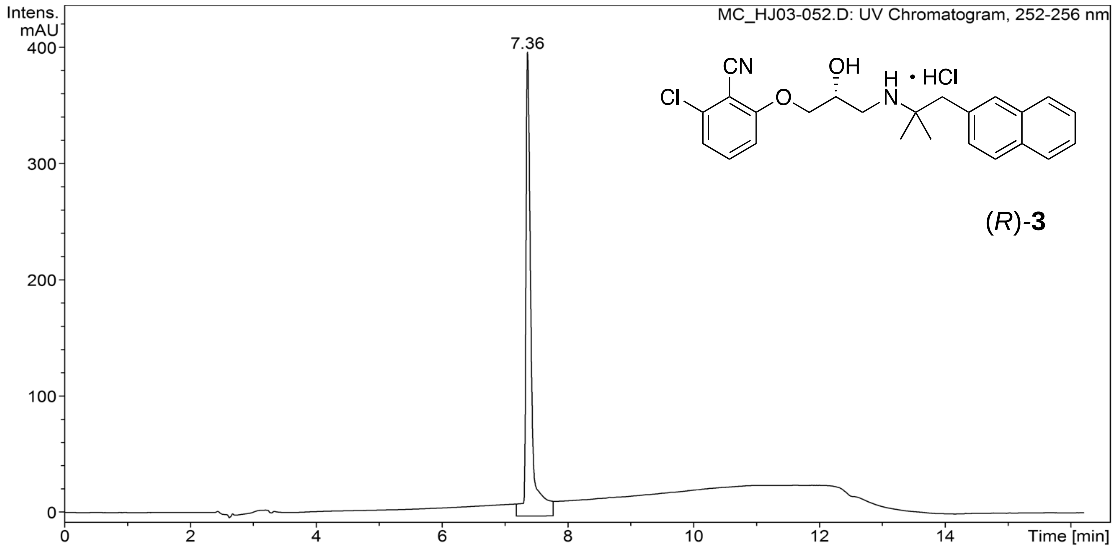

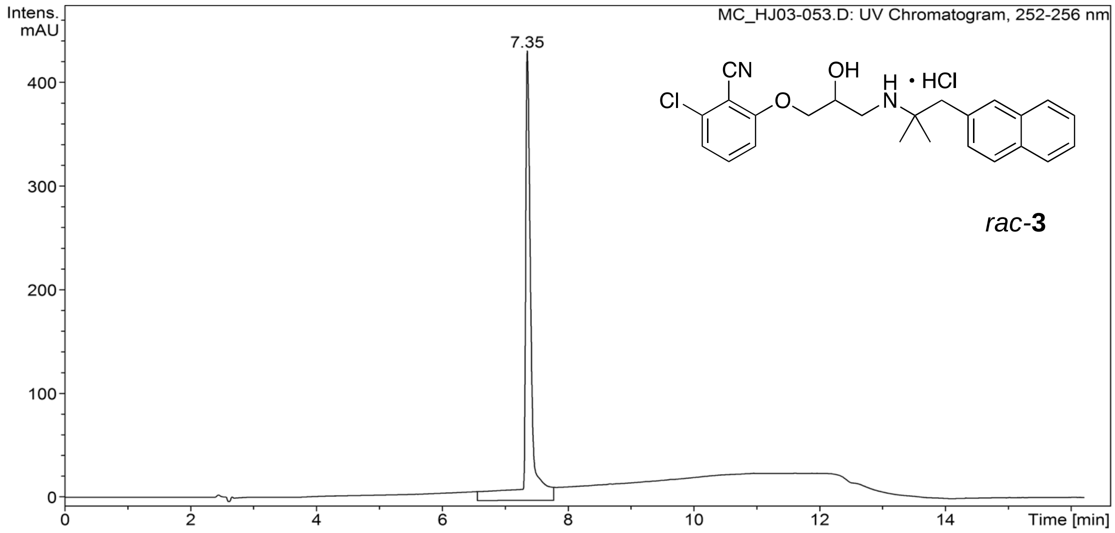

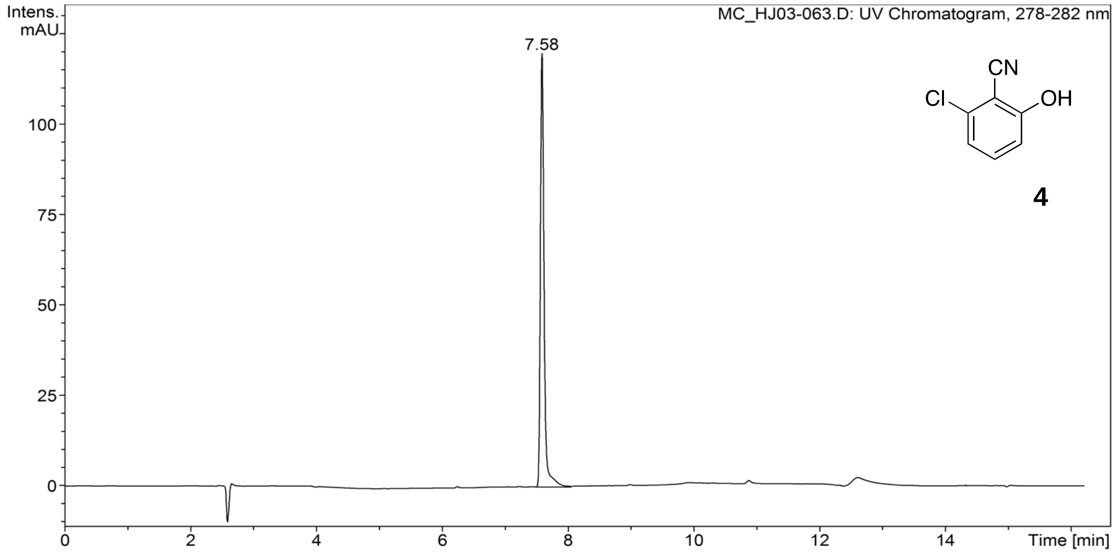

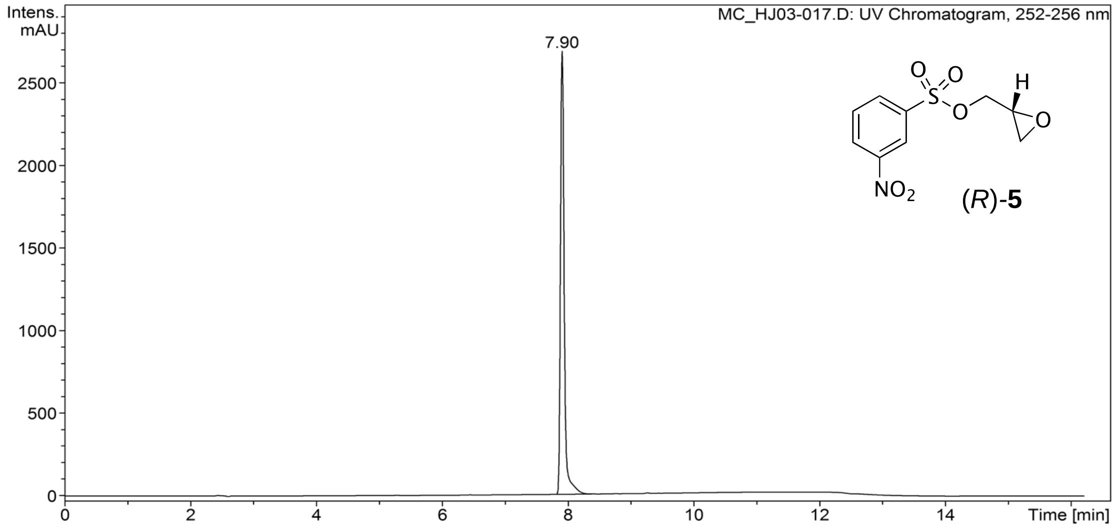
Intens.
mAU
MC_HJ03-017.D: UV Chromatogram, 252-256 nm
7.90
Time [min]
(R)-5

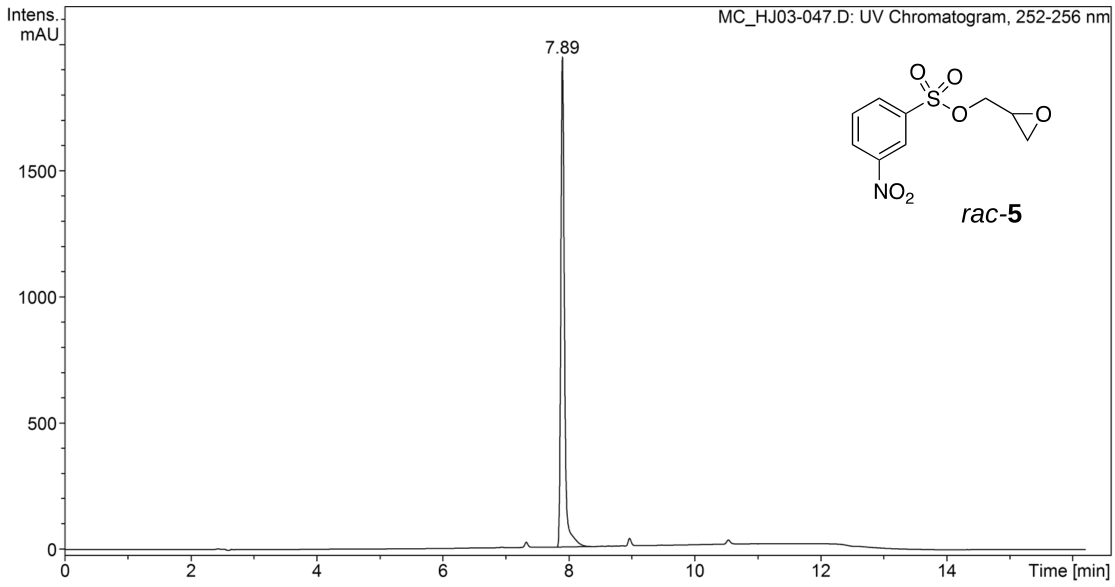
Intens.
mAU
MC_HJ03-047.D: UV Chromatogram, 252-256 nm
7.89
Time [min]
rac-5

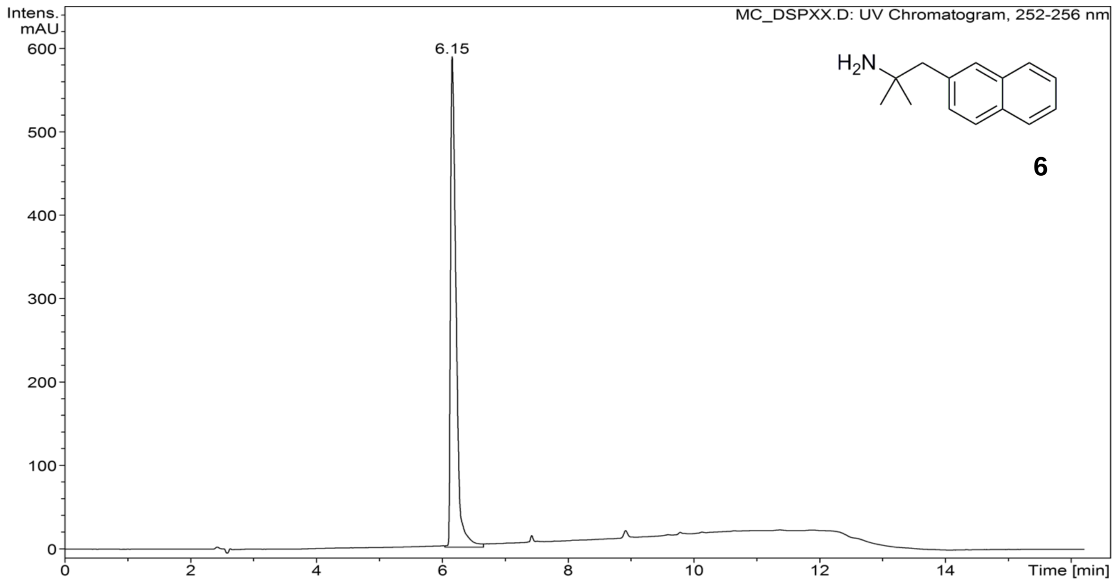
Intens.
mAU
MC_DSPXX.D: UV Chromatogram, 252-256 nm
6.15
Time [min]
6

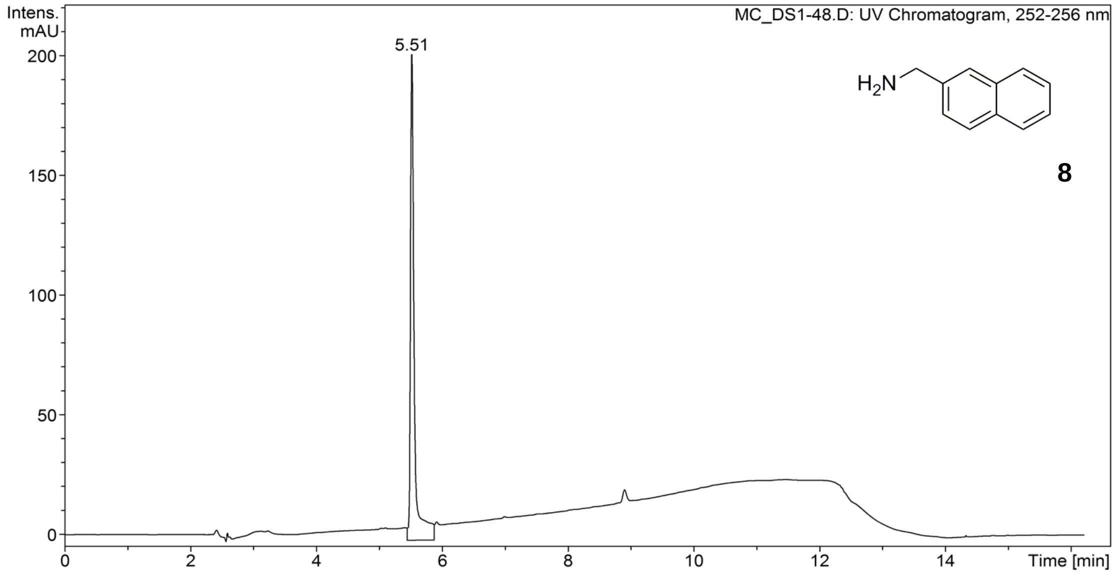
Intens.
mAU
MC_DS1-48.D: UV Chromatogram, 252-256 nm
5.51
H_2N
8
200
150
100
50
0
0 2 4 6 8 10 12 14 Time [min]

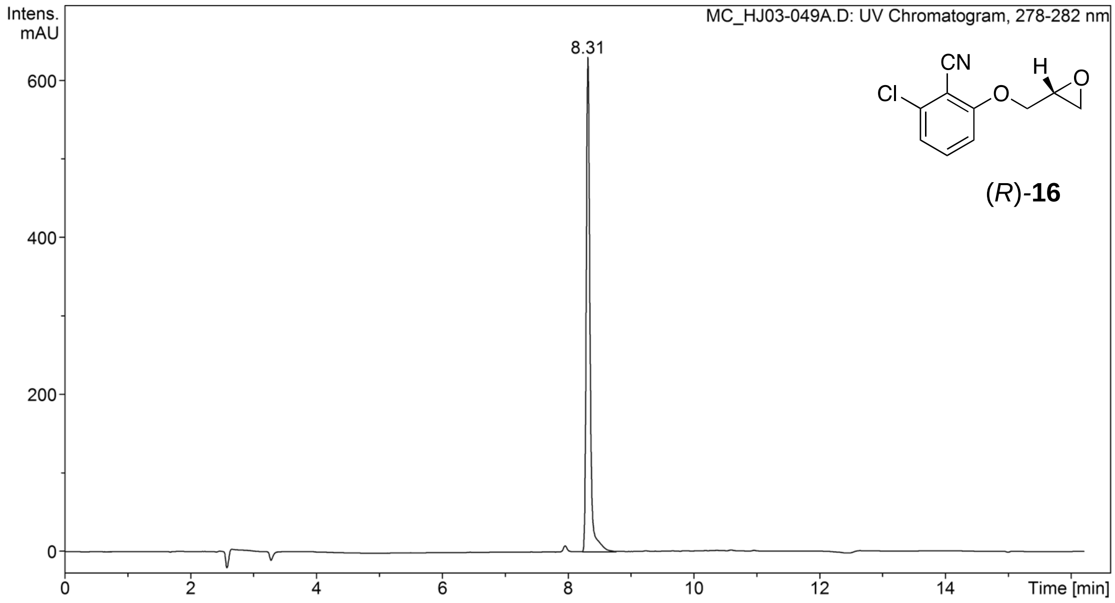
Intens.
mAU
MC_HJ03-049A.D: UV Chromatogram, 278-282 nm
8.31
CN
H
O
Cl
O
(R)-16
600
400
200
0
0 2 4 6 8 10 12 14 Time [min]

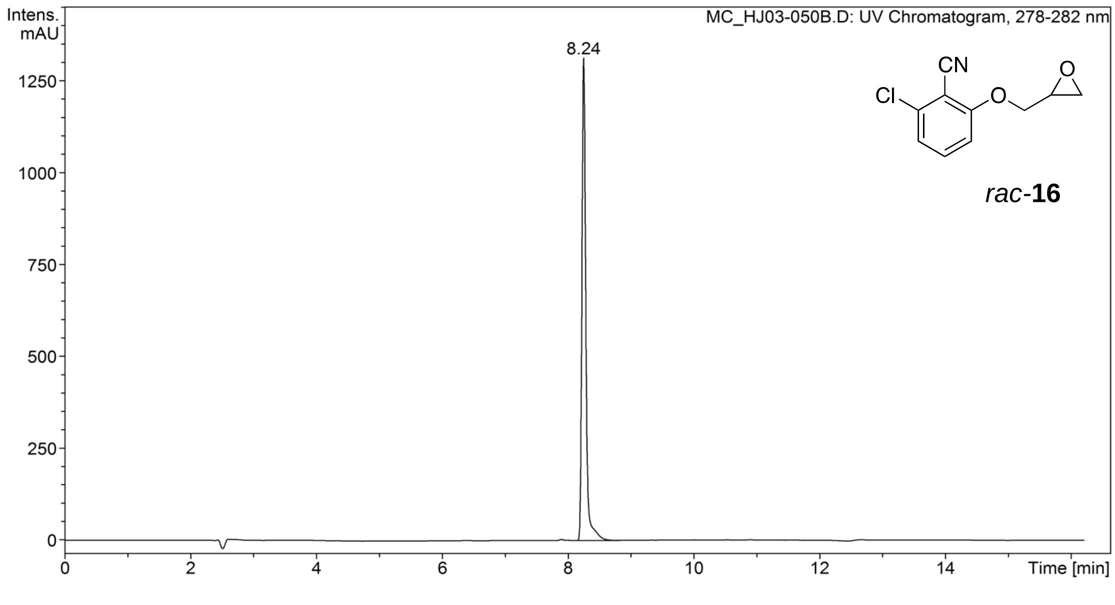
Intens.
mAU
MC_HJ03-050B.D: UV Chromatogram, 278-282 nm
8.24
CN
O
Cl
O
rac-16
1250
1000
750
500
250
0
0 2 4 6 8 10 12 14 Time [min]

Determination of optical purity

Racemic NPS 2143 (*rac*-**3**) and optically active (*R*)-**3** were subject to analysis by chiral HPLC using a Daicel Chiralpak AD-H column eluting with 30% EtOH and 0.05% diethylamine in *n*-heptane (v/v) with UV-detection at 210 nm. Integration of peaks showed an enantiomeric ratio >99:1 for (*R*)-**3** (no (*S*)-enantiomer could be detected). Optically pure (*R*)-**3** was spiked with a small amount of *rac*-**3** to confirm peak identity (see below).

26 HJ03-052_fresh

EtOH-Hep 30:70 + 0.05 DEA

Sample Name:	**HJ03-052_fresh**	*Injection Volume:*	**30,0**
Vial Number:	**GB13**	*Channel:*	**UV_VIS_1**
Sample Type:	**unknown**	*Wavelength:*	**210**
Control Program:	**20 min-1,0A-PDA_210-220-254-280**	*Bandwidth:*	**1**
Quantif. Method:	**Test METH**	*Dilution Factor:*	**1,0000**
Recording Time:	**9-1-2013 13:06**	*Sample Weight:*	**1,0000**
Run Time (min):	**20,00**	*Sample Amount:*	**1,0000**

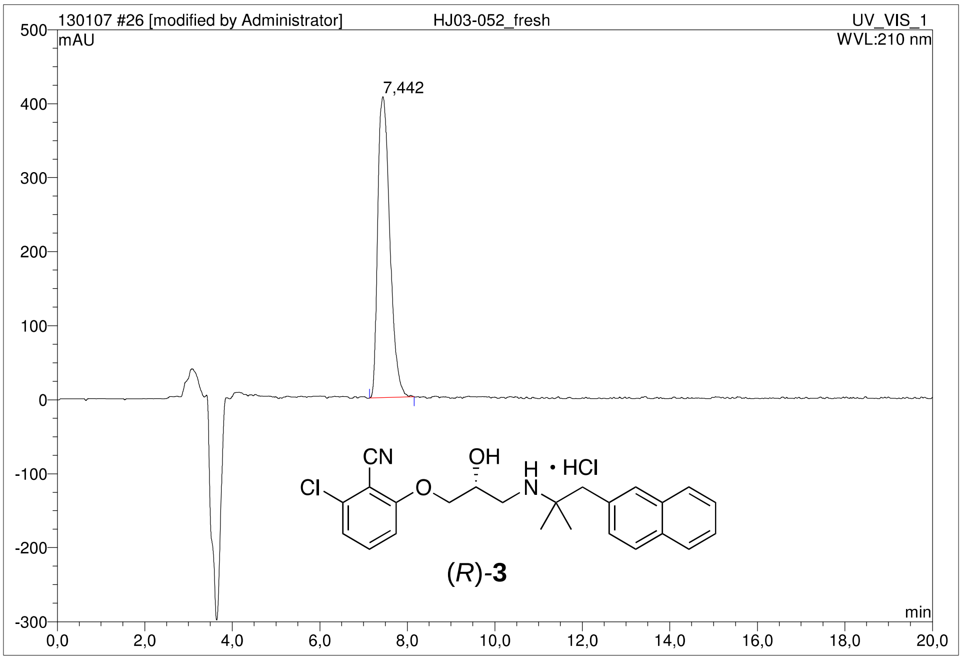

(*R*)-**3**

No.	Ret.Time min	Peak Name	Height mAU	Area mAU*min	Rel.Area %	Amount	Type
1	7,44	n.a.	406,898	130,373	100,00	n.a.	BMB*
**Total:**			406,898	130,373	100,00	0,000	

24 HJ03-053

EtOH-Hep 30:70 + 0.05 DEA

Sample Name:	**HJ03-053**	*Injection Volume:*	**30,0**
Vial Number:	**GB11**	*Channel:*	**UV_VIS_1**
Sample Type:	**unknown**	*Wavelength:*	**210**
Control Program:	**20 min-1,0A-PDA_210-220-254-280**	*Bandwidth:*	**1**
Quantif. Method:	**Test METH**	*Dilution Factor:*	**1,0000**
Recording Time:	**9-1-2013 12:08**	*Sample Weight:*	**1,0000**
Run Time (min):	**20,00**	*Sample Amount:*	**1,0000**

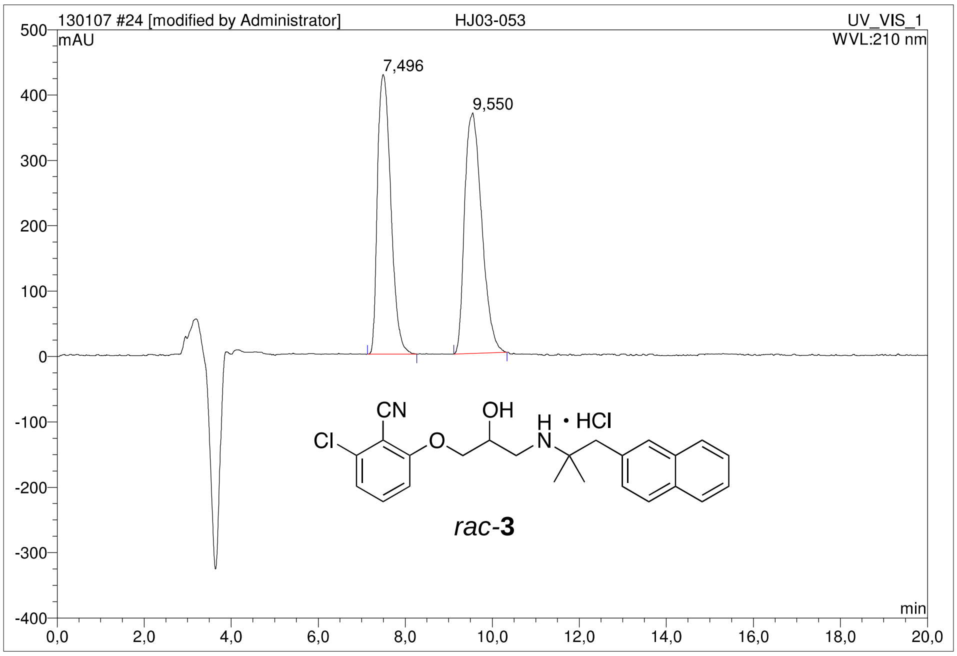

No.	Ret.Time min	Peak Name	Height mAU	Area mAU*min	Rel.Area %	Amount	Type
1	7,50	n.a.	427,803	151,511	47,52	n.a.	BMB*
2	9,55	n.a.	368,163	167,355	52,48	n.a.	BMB*
**Total:**			795,967	318,866	100,00	0,000	

27 HJ03-052spiked

EtOH-Hep 30:70 + 0.05 DEA

Sample Name:	**HJ03-052spiked**	*Injection Volume:*	**20,0**
Vial Number:	**GC1**	*Channel:*	**UV_VIS_1**
Sample Type:	**unknown**	*Wavelength:*	**210**
Control Program:	**20 min-1,0A-PDA_210-220-254-280**	*Bandwidth:*	**1**
Quantif. Method:	**Test METH**	*Dilution Factor:*	**1,0000**
Recording Time:	**9-1-2013 13:28**	*Sample Weight:*	**1,0000**
Run Time (min):	**20,00**	*Sample Amount:*	**1,0000**

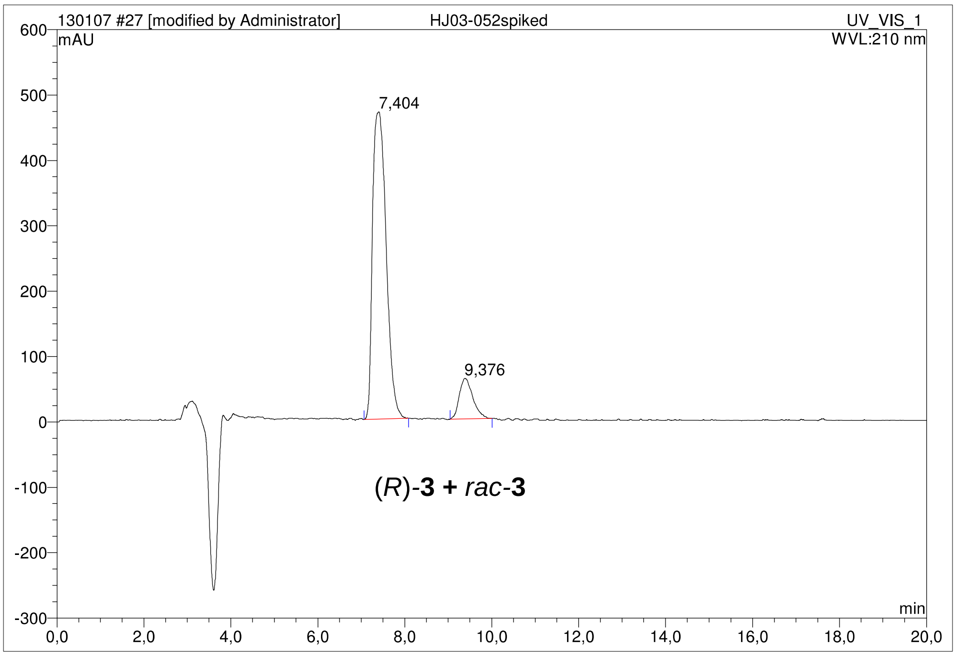

No.	Ret.Time min	Peak Name	Height mAU	Area mAU*min	Rel.Area %	Amount	Type
1	7,40	n.a.	470,106	170,433	88,37	n.a.	BMB*
2	9,38	n.a.	61,898	22,440	11,63	n.a.	BMB*
**Total:**			532,004	192,873	100,00	0,000	

Pharmacology

IP-One Assay

For the pharmacological studies a HEK293 cell line stably transfected with rat CaSR (HEK293-CaSR) was used, which had previously been characterised [5]. On the day of the experiment subconfluent stably transfected HEK293-CaSR cells were washed one time with DPBS and detached from the cell culture plate using dissociation buffer (Sigma-Aldrich, St Louis, MO, USA). Cells were centrifuged and resuspended in assay buffer (20 mM HEPES + 0.1% BSA in HBSS buffer, pH 7.4) at a concentration of 1,000,000 cells/mL. In a 384 well OptiPlate (PerkinElmer, Waltham, MA, USA), 2 µL of ligand buffer containing NPS 2143 ((*R*)-**3**) and 3.5 mM $CaCl_2$ was then mixed with 10 µL of cell suspension. The plate was sealed and incubated at 37 °C for 1 h, followed by 15 min incubation at room temperature. Next, 10 µL of detection reagents (lysis buffer containing 2.5% Eu^{3+}-anti-IP_1 antibody and 2.5% IP_1-d2) was added and the plate was incubated for 1 h at room temperature. The plate was read on an Envision (PerkinElmer, Waltham, MA, USA) where the wells were excited with light at 340 nm, and the emitted light was measured at 615 nm and 665 nm. The time resolved-fluorescence resonance energy transfer (TR-FRET) 665 nm/615 nm ratio was used to calculate IP_1 concentrations from a standard curve generated by IP_1 standards provided by the manufacturer (Cisbio, Bagnols, France).

References

1. Sejer Pedersen, D.; Rosenbohm, C. *Synthesis* **2001,** 2431–2434. doi:10.1055/s-2001-18722
2. Marquis, R. W.; Lago, A. M.; Callahan, J. F.; Trout, R. E. L.; Gowen, M.; DelMar, E. G.; Van Wagenen, B. C.; Logan, S.; Shimizu, S.; Fox, J.; Nemeth, E. F.; Yang, Z.; Roethke, T.; Smith, B. R.; Ward, K. W.; Lee, J.; Keenan, R. M.; Bhatnagar, P. *J. Med. Chem.* **2009,** *52,* 3982–3993. doi:10.1021/jm900364m
3. Fagerström, A.; Nilsson, M.; Berg, U.; Isaksson, R. *Org. Biomol. Chem.* **2006,** *4,* 3067–3076. doi:10.1039/b605603b
4. Katritzky, A. R.; De Ville, G.; Patel, R. C. *Tetrahedron* **1981,** *37,* 25–30. doi:10.1016/0040-4020(81)85037-5
5. Thomsen, A. R. B.; Hvidtfeldt, M.; Bräuner-Osborne, H. *Cell Calcium* **2012,** *51,* 107–116. doi:10.1016/j.ceca.2011.11.009
